# Cell-free 3D scaffold with two-stage delivery of miRNA-26a to regenerate critical-sized bone defects

**DOI:** 10.1038/ncomms10376

**Published:** 2016-01-14

**Authors:** Xiaojin Zhang, Yan Li, Y. Eugene Chen, Jihua Chen, Peter X. Ma

**Affiliations:** 1 Department of Biologic and Materials Science, University of Michigan, Ann Arbor, Michigan 48109, USA; 2 State Key Laboratory of Military Stomatology, Department of Prosthodontics, School of Stomatology, The Fourth Military Medical University, Xi'an 710032, China; 3 Department of Cardiac Surgery, Frankel Cardiovascular Center, University of Michigan, Ann Arbor, Michigan 48109, USA; 4 Department of Biomedical Engineering, University of Michigan, Ann Arbor, Michigan 48109, USA; 5 Macromolecular Science and Engineering Center, University of Michigan, Ann Arbor, Michigan 48109, USA; 6 Department of Materials Science and Engineering, University of Michigan, Ann Arbor, Michigan 48109, USA

## Abstract

MicroRNAs (miRNAs) are being developed to enhance tissue regeneration. Here we show that a hyperbranched polymer with high miRNA-binding affinity and negligible cytotoxicity can self-assemble into nano-sized polyplexes with a ‘double-shell’ miRNA distribution and high transfection efficiency. These polyplexes are encapsulated in biodegradable microspheres to enable controllable two-stage (polyplexes and miRNA) delivery. The microspheres are attached to cell-free nanofibrous polymer scaffolds that spatially control the release of miR-26a. This technology is used to regenerate critical-sized bone defects in osteoporotic mice by targeting *Gsk-3β* to activate the osteoblastic activity of endogenous stem cells, thus addressing a critical challenge in regenerative medicine of achieving cell-free scaffold-based miRNA therapy for tissue engineering.

## Abstract (web summary)

A challenge in regenerative medicine is the development of cell-free, non-immunogenic miRNA-delivering scaffolds. Here the authors design a cell-free scaffold capable of efficient and prolonged delivery of miRNA-26a to endogenous cells and show that it can regenerate a full-thickness calvarial bone defect in mice.

## Graphical abstract

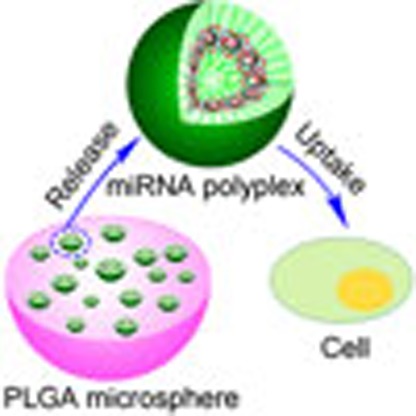

MicroRNAs (miRNAs) are endogenous small non-coding RNAs that function as post-transcriptional repressors by binding to partially complementary sites on target messenger RNAs (mRNAs)^1^. Owing to coulomb repulsion, miRNAs and their mimics or inhibitors, which are negatively charged, cannot easily cross the cell membrane, which is also negatively charged^2^. In addition, ‘naked’ miRNAs are rapidly degraded *in vivo*^3^. Therefore, vectors that protect and deliver miRNAs into cells are required for miRNA therapy. Although viral vectors can deliver miRNAs efficiently into cells^4^,^5^,^6^, immune responses to these viral vectors are of concern^7^. Lipofectamine is a commercial non-viral vector that forms liposome vesicles to encapsulate RNAs (called lipoplexes) and transport them into cells *in vitro*^8^. However, *in vivo* the transfection efficiency of lipoplexes is low, possibly because liposomes are unstable in the blood^9^. The stability and transfection efficiency of miRNA mimics or their inhibiting antisense oligoribonucleotides can be improved^10^. For example, cholesterol has been linked to oligoribonucleotides to enhance transfection efficacy^11^. However, each miRNA or its inhibitor needs to be modified specifically to form an ‘agomir’ or ‘antagomir’. Polymer vectors are less immunogenic than viral vectors and are inexpensive, and have, therefore, been developed as carriers of DNAs and short interfering RNAs (siRNAs)^12^,^13^,^14^,^15^,^16^,^17^. However, polymeric vectors for therapeutic miRNAs often have low transfection efficiency. Polyethylenimine (PEI) is the most widely studied polymer for DNA delivery and has been used to deliver miR-145 and miR-33a in colon carcinoma^18^ and lung tumour^19^ mouse models. However, PEI with a high molecular weight is toxic to cells, and PEI with a low molecular weight has low transfection efficiency^20^. Although the toxicity of high molecular weight PEI is not a major concern in the context of cancer therapy, it would be a major concern for regenerative therapy.

Overcoming the need for the addition of cells to scaffolds is a critical challenge in the field of tissue engineering; ideally, endogenous cells would be utilized^21^,^22^. Here we show that sustained local miRNA delivery can activate endogenous stem and progenitor cells to regenerate critical-sized tissue defects. We design a hyperbranched polymer (HP) vector for miRNA delivery, in which short polyethylene glycol (PEG) chains and a low molecular weight cationic PEI are attached to the outer shell of a hyperbranched hydrophobic molecular core. Addition of miRNA causes further self-assembly into a nano-sized spherical shell sandwiched between the inner and outer hydrophilic PEG layers. To overcome problems with uncontrolled release associated with current miRNA delivery systems, these stable polyplexes carrying miR-26a are encapsulated in biodegradable polymer microspheres (MS). This two-stage miRNA delivery strategy enables both controllable duration (first stage, MS) and high transfection efficiency (second stage, polyplexes). Furthermore, to prevent off-target effects of the miRNA delivery, we immobilize the MS on a nanofibrous (NF) cell-free three-dimensional (3D) scaffold to spatially and temporally control activation of endogenous cells and regenerate critical-sized calvarial bone defects in healthy and osteoporotic mice (Fig. 1).

## Results

### Polymer synthesis

**Figure 2:**
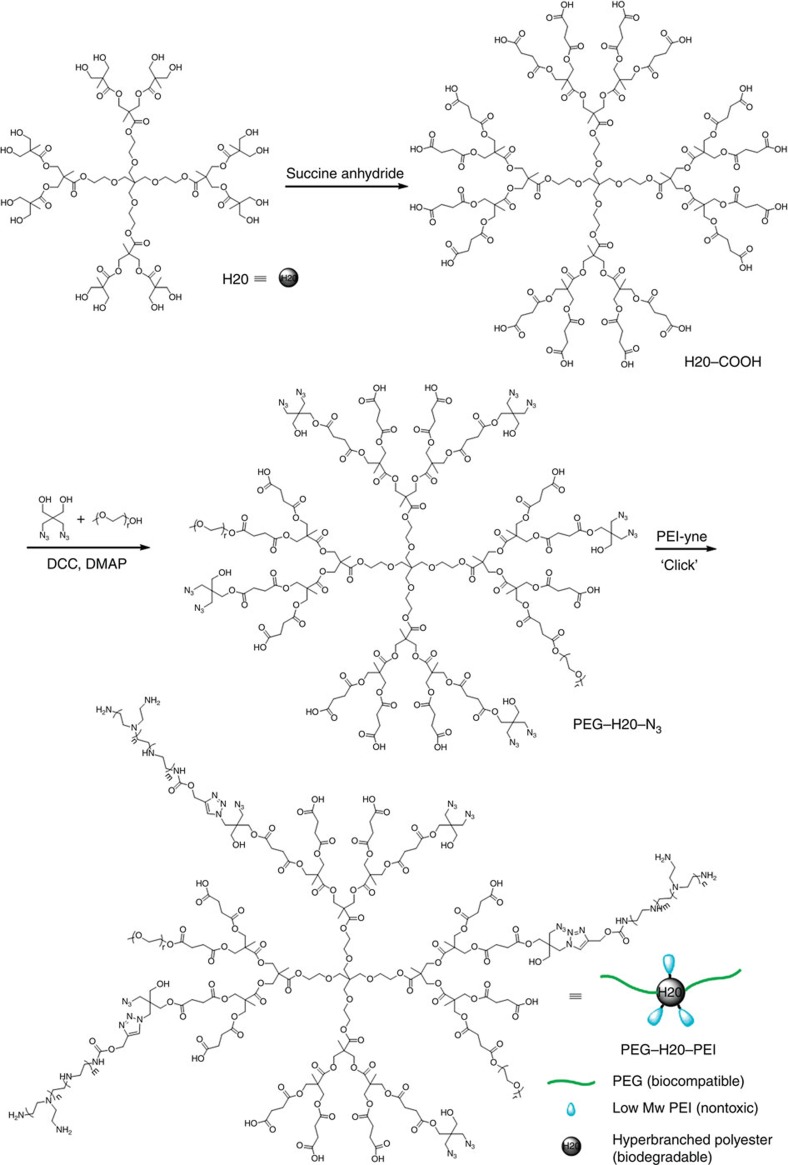
Synthesis of hyperbranched PEG–H20–PEI co-polymer. First, the hydroxyl groups of hyperbranched polyester (H20) were converted into carboxyl groups through succinic anhydride at room temperature. Then, the selected PEG chains reacted with a carboxyl group through condensation to attach on the hyperbranched bis-MPA polyester core. At the same time, certain azido groups also reacted with the carboxyl groups on the hyperbranched bis-MPA polyester. Then, PEI-yne reacted with azido groups on the hyperbranched bis-MPA polyester via a click reaction.

**Figure 3:**
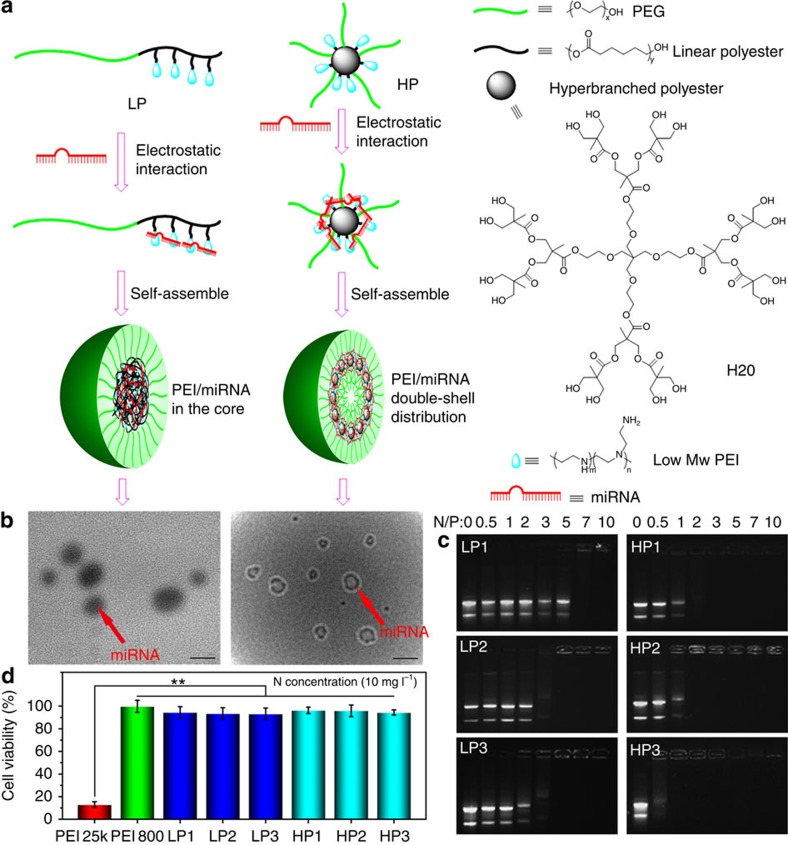
The polyplex formation and characterization. The polymers and miRNA formed the polyplexes via the electrostatic interaction and self-assembly (**a**). The polyplexes were characterized by TEM (scale bar, 100 nm) (**b**). Here we established a novel negative staining method for TEM study. Only miRNA was negatively stained with tungstic acid. For linear polymers, the polymer/miRNA polyplexes were solid spheres. However, miRNA was found with a double spherical shell distribution—miRNA assembled on the outer shell of HP molecular core and HP/miRNA polyplexes formed a larger spherical shell sandwiched between the outer and inner PEG layers. The binding affinities of the polymers with miRNA were evaluated using agarose gel electrophoresis (**c**). When a cationic polymer and the miRNA form a stable polyplex with positive surface potential, the miRNA band would stay in the original position after being electrophoresed, that is, the cationic polymer would retard miRNA migration. The results indicated that hyperbranched polymers retarded miRNA migration at lower N/P ratios than linear polymers. The cytotoxicity assay of polymers was carried out using an MTT assay (**d**). PEI 25 kDa had a very high toxicity, while PEI 800 was nontoxic. PEI 800 was chosen as the cationic compound to be conjugated to polymers in this work. After PEI conjugation, both the linear polymers and the hyperbranched polymers had a very low cytotoxicity. **P<0.01; *n*=5 per group. Data are mean±s.d.

We designed two groups of biodegradable polymers (linear and hyperbranched polyesters, abbreviated as LPs and HPs, respectively) and three different molecular weights for each group. Low molecular weight PEI (average molecular weight, 800 Da) and defined PEG chains were attached on the synthesized polyesters with click chemistries (Fig. 2, Supplementary Figs 1–4 and Supplementary Table 1). The hydrophilic PEG is biocompatible and can form a stealth layer^23^,^24^,^25^ to protect the inner polymer/miRNA polyplexes (Fig. 3a). The low molecular weight PEI is non-toxic^26^.

### Polymer/miRNA polyplexes

The polymer/miRNA polyplexes were prepared by incubating a mixture of polymer and miRNA at room temperature for 30 min. Utilizing the coupling interactions between phosphorus (P) in miRNA and tungsten (W) in tungstic acid^27^, the miRNA was labelled with freshly prepared tungstic acid (see Methods). The transmission electron microscopy (TEM) images of LP/miRNA polyplexes and the HP/miRNA polyplexes were clearly different (Fig. 3b). The dark miRNA in the LP/miRNA polyplexes aggregated into spherical cores. However, for the HPs, the miRNA first complexed with the PEI on the outer shell of the hyperbranched polyester cores. The HP molecular cores and PEI/miRNA shells together assembled into the hydrophobic spherical shell sandwiched between the inner PEG core and the outer PEG chains of the three-layer nanospheres (dark circles under TEM). Under our experimental conditions, the thickness of the outer layer and the radius of the inner core were found to be on the same order of magnitude, approximately the theoretical length of the PEG chains (Supplementary Fig. 5). Without miRNA, the HPs self-assemble into polymeric micelles in water (for example, HP3 has a diameter of ∼40 nm measured using dynamic light scattering). However, on addition of miRNA, the formed HPs/miRNA polyplexes have a significantly larger particle size (for example, ∼152 nm for HP3/miR-26a measured using dynamic light scattering (DLS)). These data once again suggest that HPs form unimolecular micelles and further self-assemble into significantly larger nano-sized polyplexes, in which the negatively charged miRNAs bind the positive charged PEI on the HPs, facilitating the polyplex formation.

**Figure 4:**
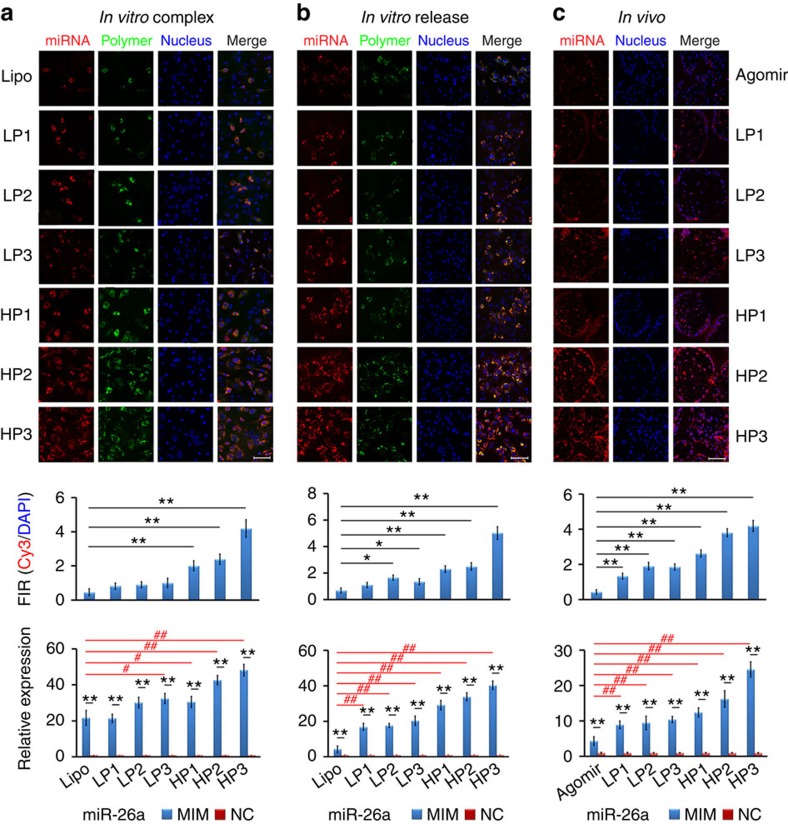
miRNA transfection efficiencies. miR-26a and miR-26a agomir were labelled using Cy3 (orange red); the polymers (vectors) were labelled using FITC (green); and the cell nuclei were stained with DAPI (blue). To label lipofectamine 2000, NBD cholesterol (10% by weight) was added into lipoplex solution. The confocal images were taken after 2 days of transfection or 2 weeks after implantation. The transfection efficiency was determined by quantifying the fluorescence intensity and the miRNA expression level. (**a**) miRNA (60 pmol) was complexed with polymers at an N/P ratio of 10 and incubated at room temperature for 30 min, or with lipofectamine 2000 following the manufacturer’s protocol, and then co-cultured with osteoblasts in the presence of 10% FBS *in vitro* for 2 days. (**b**) The polymer/miRNA polyplexes were released from the PLGA microspheres immobilized on NF PLLA scaffolds and co-cultured with osteoblasts *in vitro* for 2 days. (**c**) The PLLA scaffolds with the immobilized PLGA microspheres containing the polymer/miRNA polyplexes or agomir were implanted on the back of nude mice *in vivo* for 2 weeks. *P<0.05; **P<0.01; #*P*<0.05; ##*P*<0.01. All experiments were carried out in triplicate. *n*=5 per group. Data are mean±s.d. Scale bar, 100 μm.

To test our hypothesis that such double-shell-like charge (PEI) distribution of HP vectors results in lower cytotoxicity, higher affinity to miRNA and higher transfection efficiency than those of LP vectors, we first examined the complexing capacities of these cationic polymers with the miRNA using agarose gel electrophoresis. The HPs had higher affinity to miRNA (retarding miRNA migration at lower polymer quantity) than LPs when they had similar molecular weights and nitrogen contents (Fig. 3c). In addition, the new HPs were shown to have substantially lower toxicities than the high molecular weight PEI (average molecular weight: 25,000 Da; Fig. 3d). HPs had substantially higher transfection efficiencies than LPs or lipofectamine 2000 *in vitro*, as evidenced by the greatest amount of miRNA polyplexes inside cells and the highest level of the miRNA expression (Fig. 4a).

### Controlled two-stage miRNA release

Then, the stabilities of the HP/miRNA polyplexes and the most widely used commercial product lipofectamine 2000/miRNA lipoplexes were examined side-by-side. Lipoplexes were larger in size with an average diameter of ∼653 nm and were unstable under sonication, resulting in a broader size distribution and a size increase to 1,341 nm because of fragmentation and aggregation (Supplementary Fig. 6). In contrast, HP/miRNA polyplexes were smaller in size with an initial average diameter of ∼224 nm and were stable under sonication, resulting in a narrower size distribution and a slightly reduced average size of 152 nm under sonication (Supplementary Fig. 7) likely due to mechanically induced denser packaging. When the ratios of PEG/polyesters were kept the same, the diameters of the HP/miRNA polyplexes were smaller than and the zeta potentials were higher than those of LP/miRNA polyplexes because of the highly concentrated branching structure and the shorter PEG chain on HPs (Supplementary Fig. 8). The zeta potentials of HP/miRNA and LP/miRNA polyplexes were higher than the low molecular weight PEI (weight average 800 Da), but lower than the high molecular weight PEI (weight average 25 kDa), increasing with increasing polyester molecular weight and degree of branching (Supplementary Fig. 8b). These data indicate that, although lipoplexes were unstable under sonication, HP/miRNA polyplexes were stable.

To achieve the first-stage controlled release of the HP/miRNA polyplexes, the HP/miRNA polyplexes were encapsulated in poly(lactic-*co*-glycolic acid) (PLGA) MS with an average diameter of ∼3 μm via a well-established double emulsion method^28^. Compared with the naked miRNA, lipofectamine/miRNA lipoplexes and the agomir (cholesterol-modified miRNA mimic), the polymer/miRNA polyplexes were encapsulated in the PLGA MS with higher efficiencies (Supplementary Figs 9 and 10). The encapsulation efficiency of lipofectamine 2000/miRNA lipoplexes in PLGA was very low, even lower than that of naked miRNA (Supplementary Fig. 10), possibly resulting from their low stability and high hydrophilicity. PLGA MS encapsulating HP/miRNA polyplexes were subsequently immobilized on a NF poly(L-lactic acid) (NF PLLA) scaffold (Supplementary Fig. 11) using a modified method for growth factor release in our laboratory^29^. The interconnectivity between pores (important for cell-seeding and migration^30^) and the NF morphology (advantageous for bone regeneration^31^) were retained after the PLGA MS immobilization. The first-stage release of miRNA polyplexes was examined in PBS. The released polyplexes remained to be nanoparticles (Supplementary Fig. 12). The MS made of PLGA with an average molecular weight of 6.5 k (PLGA6.5k) was chosen for a shorter-term release, while PLGA with an average molecular weight of 64 k (PLGA64k) was chosen for a longer-term release. After burst release of either LP/miRNA or HP/miRNA polyplexes during the first day, there was sustained release of the polyplexes from the PLGA6.5k MS for ∼2 weeks. In contrast, after burst release of polyplexes during the first day, there was sustained release of the polyplexes from the PLGA64k MS for longer than a month (Supplementary Fig. 13).

The released LP/miRNA or HP/miRNA polyplex solution was incubated with osteoblasts (Obs) for 48 h to examine the second-stage delivery, that is, the delivery of miRNA into cells by the polyplexes. The confocal images and the fluorescence intensity quantification showed that HP/miRNA polyplexes had higher transfection efficiencies and resulted in higher miRNA expression inside cells than LP/miRNA polyplexes and lipofectamine 2000 (Fig. 4b). The PLLA scaffold was subcutaneously implanted into mice. HP series were more effective than LP series and the agomir in transfecting cells *in vivo* (Fig. 4c).

### Ectopic bone formation

**Figure 5:**
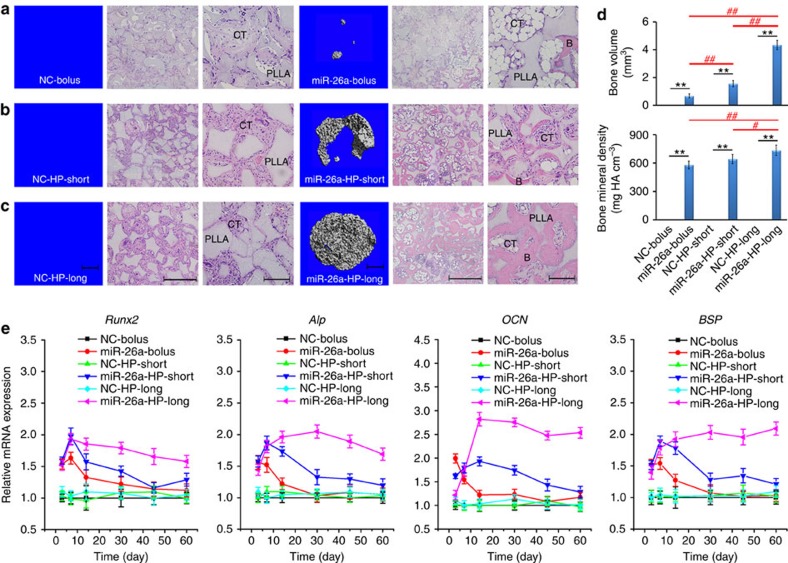
New bone formation in a subcutaneous implantation model. Continuous high-efficiency delivery of miR-26a significantly increases ectopically formed bone volume *in vivo* through elevating the expression levels of the key osteogenic factors for a prolonged time. Polymer HP3/miR-26a or NC polyplexes (miR-26a-bolus/NC-bolus) (**a**), HP3/miR-26a or NC polyplex-loaded PLGA 6.5-K microspheres (miR-26a-HP-short/NC-HP-short) (**b**) and HP3/miR-26a or NC polyplex-loaded PLGA 64-K microspheres (miR-26a-HP-long/NC-HP-long) (**c**) were attached directly to PLLA scaffolds, seeded with ∼1 × 10^5^ bone marrow mesenchymal stem cells, and subcutaneously implanted into immunocompromised mice for 2 months. MicroCT images (left) and haematoxylin and eosine staining images (right) of the implants are reported. Formation of bone (B) and connective tissue (CT) around the PLLA scaffolds are highlighted (**c**). The new bone volume and bone mineral density from these groups are quantified (**d**). (**e**) Pattern and time course changes of early-stage and late-stage osteogenic genes in the implants (including *Runx2, Alp, OCN* and *BSP*) among the six groups from days 3 to 60 were determined with real-time RT–PCR. **P<0.01; #*P*<0.05; ##*P*<0.01. All experiments were performed in triplicate. *n*=5 per group. Data are mean±s.d. Scale bars, 1 mm (in microCT images), 500 μm (in haematoxylin and eosin (H&E) images at right), 200 μm (in higher-magnification H&E images at far right).

A well-established subcutaneous implantation model was used to test whether the local two-stage delivery system that we developed could sustain the delivery of miR-26a into cells *in vivo* for a prolonged time and retain the ability to upregulate the expression of key osteogenic factors at a therapeutic level, increasing new bone volume. Considering that the cell-seeding density significantly affects bone regeneration capacity in a cell-seeded scaffold^32^, a low cell (mouse mesenchymal stem cell)-seeding density of 1 × 10^5^ cells per scaffold was selected as there was no appreciable new bone volume in all groups delivered with negative control (NC) for the miRNA (based on microCT imaging and haematoxylin and eosine staining evaluations). In contrast, there was appreciable new bone volume in all miR-26a delivery groups, with the highest amount of new bone volume with the long-term miR-26a delivery group, lower amounts of new bone volume with the short-term miR-26a delivery group and the lowest amount of new bone volume with the bolus miR-26a delivery group (Fig. 5a–c). Quantitative microCT analysis shows that the new bone volume in the long-term miR-26a delivery group was two times higher than that in the short-term miR-26a delivery group, and six times higher than that in the bolus miR-26a delivery group (Fig. 5d).

We monitored the mRNA expression of both early-stage osteogenic makers (Runt-related transcription factor 2 (*Runx2*) and alkaline phosphatase (*Alp*)) and the late-stage mineralization markers (osteocalcin (*OCN*) and bone sialoprotein (*BSP*)) at six selected time points during the 60-day implantation. As shown in Fig. 5e, the four key osteogenic markers showed similar mRNA expression profiles among the six groups during the time course. No significant changes in mRNA expression levels were observed in all the NC delivery groups. In contrast, *Runx2*, *Alp*, *OCN* and *BSP* mRNA expression levels were enhanced in the miR-26a delivery groups at early stages (days 3 and 7), followed by robust expression in the long-term miR-26a delivery group, lower expression in the short-term delivery group and the lowest level of expression in the bolus miR-26a delivery group during the matrix maturation period (day 14). During the late mineralization stage (days 30–60), there was a continued decline in the mRNA level of these osteogenic markers in the short-term miR-26a delivery group. However, the mRNA expression of these four markers was sustained at high levels in the long-term miR-26a delivery group. These results indicate that miR-26a upregulates multiple osteogenic factors at various osteoblastic differentiation stages to enhance bone formation, and the long-term continuous delivery of miR-26a most significantly enhance bone formation.

### Critical-sized calvarial bone-defect repair

**Figure 6:**
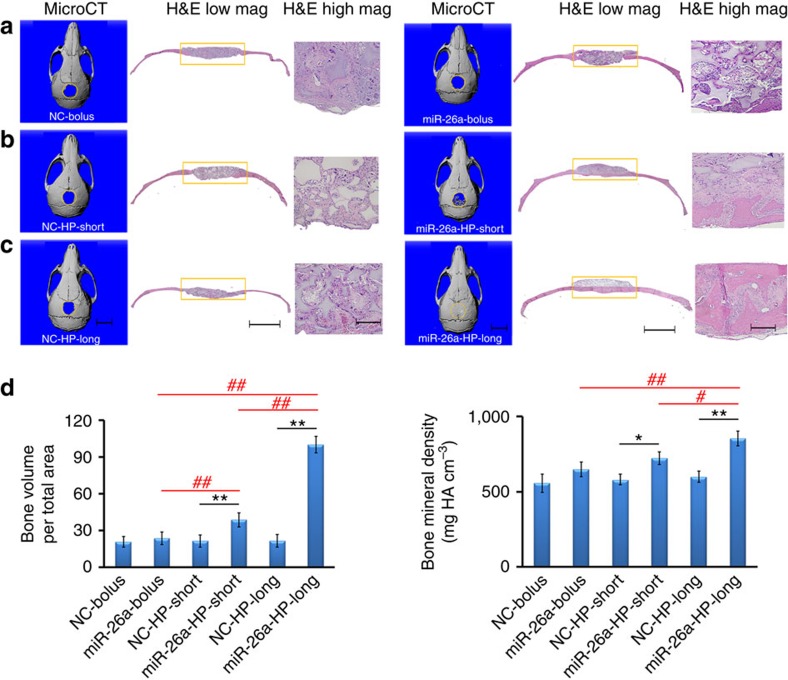
Critical-sized calvarial bone-defect repair. (**a**) Cell-free NF PLLA scaffolds were directly coated with HP/miR-26a polypexes or the NC polyplexes (miR-26a-bolus or NC-bolus). (**b**) Cell-free NF PLLA scaffolds were immobilized with PLGA 6.5-K microspheres that contains HP/miR-26a polyplexes or NC polyplexes (miR-26a-HP-short or NC-HP-short). (**c**) Cell-free NF PLLA scaffolds were immobilized with PLGA 64-K microspheres that contains HP/miR-26a polyplexes or NC polyplexes (miR-26a-HP-long or NC-HP-long). The above six groups of scaffolds were implanted into 5-mm critical-sized calvarial bone defects of C57BL/6d mice. MicroCT images (left) and H&E staining (low magnification (mag) in the middle and high mag on the right) of calvarial defects were recorded after implantation for 2 months. (**d**) Quantitative microCT analysis showing the regenerated bone volumes and bone mineral densities in the six groups described above. Consistent with significantly increased neo bone volumes, the bone mineral densities of the short-term and long-term miR-26a release groups were also increased. *P<0.05; **P<0.01; #*P*<0.05; ##*P*<0.01. All experiments were carried out in triplicate. *n*=5 per group. Data are mean±s.d. Scale bars, 5 mm (in microCT images), 2.0 mm (in H&E images at right), 200 μm (in higher-mag H&E images at far right).

Then, cell-free, two-stage miR-26a-delivering scaffold was used to fill a critical-sized calvarial bone defect in C57BL/6 mice. Eight weeks later, there was minimal new bone volume in all NC delivery groups (Fig. 6); there was a slightly increased new bone volume but not at a statistically significant level in the bolus miR 26a delivery group; there was increased new bone volume at a statistically significant level in the short-term miR-26a delivery group; and, excitingly, the bone defect was completely repaired in the long-term miR-26a delivery group.

To understand how the *in situ* sustained miR-26a delivery enhanced bone regeneration, we monitored the mRNA expression levels of the early-stage osteogenic markers (*Runx2* and *Alp*) and the mineralization-stage markers (*OCN* and *BSP*) over time (Supplementary Fig. 14). In the early stage, *Runx2*, *Alp*, *OCN* and *BSP* increased in the miR-26a delivery groups. The *in situ* long-term miR-26a delivery enhanced the expression of all examined osteogenic genes and especially the mineralization-stage genes (*OCN* and *BSP*) at higher levels for a prolonged time, which appeared to be critical in enhancing the osteogenic differentiation of the recruited cells and thereby the progressive bone regeneration throughout. Implantation time-wise, there was substantial bone regeneration on the bottom side of the defect (dura side) at 5 weeks and progressively extended throughout the entire defect at 8 weeks (Supplementary Fig. 15), suggesting that the stem cells for bone regeneration were from the dura. The porous NF PLLA scaffold precisely defined the 3D defect regeneration and facilitated cell migration and uniform distribution of recruited cells^31^,^33^.

### Critical-sized calvarial defect repair in osteoporotic mice

**Figure 7:**
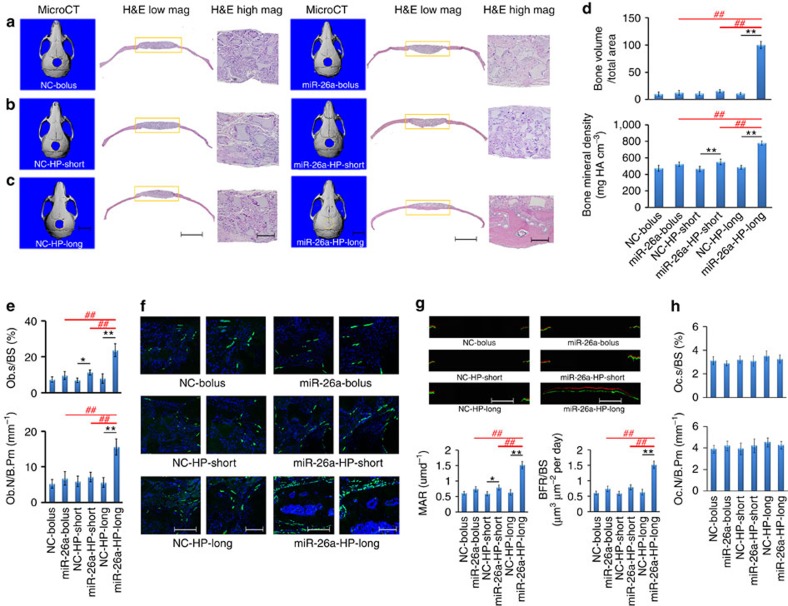
Critical-sized calvarial bone-defect repair in overiectomy-induced osteoporotic (OVX) mice. (**a**) Cell-free PLLA scaffolds with physically coated HP/mi-R26a or NC polyplexes (miR-26a-bolus or NC-bolus), (**b**) cell-free PLLA scaffolds with immobilized PLGA 6.5-K microspheres loaded with HP/miR-26a or NC polyplexes (miR-26a-HP-short or NC-HP-short) and (**c**) cell-free PLLA scaffolds with immobilized PLGA 64-K microspheres loaded with HP/miR-26a or NC polyplexes (miR-26a-HP-long or NC-HP-long) were implanted in 5-mm critical-sized calvarial bone defects in OVX mice. MicroCT images (left) and H&E staining (middle and right) of calvarial defects were recorded after implantation for 2 months. Scale bars, 5 mm in microCT images, 2.0 mm in H&E images in the middle, 200 μm in the high-mag H&E images on the right. (**d**) Quantitative analysis showing the new bone volumes and new bone mineral densities in the above-described groups. (**e**) Osteoblast surface/bone surface ratios and osteoblast number/bone perimeter ratios in OVX mice under the above-described groups. (**f**) Immunofluorescence staining for OCN (green) and DAPI staining for nuclei (blue) in histological sections of the healing calvarial bone defects in OVX mice in the above-described groups, where the scale bars, 500 μm (left), 200 μm (right). (**g**) New bone-formation front in the calvarial bone defect was sequentially labelled with fluorescent calcein and xylenol in OVX mice in the six groups. Representative fluorescent micrographs show the xylenol (red) and calcein (green) bands, where the scale bars, 2 mm. (**h**) The Oc.S/BS and Oc.N/B.Pm were determined using tartrate-resistant acid phosphatase (TRAP) staining. *P<0.05; **P<0.01; #*P*<0.05; ##*P*<0.01. All experiments were performed in triplicate. *n*=5 per group. Data are mean±s.d.

We hypothesized that long-term miRNA delivery could overcome impaired bone-healing capacity of osteoporotic mice because of the resulting prolonged high-level expression of osteogenic marker genes. To test this hypothesis, the osteoporotic mice were established 2 months after ovariectomizing (OVX) female C57BL/6J mice^34^ (Supplementary Note 1 and Supplementary Figs 16–18). Their MSCs and Obs were found to have attenuated osteogenic capacity (Supplementary Note 1 and Supplementary Figs 19 and 20). As miR-26a treatment rescued attenuated bone-formation capacity of OVX–MSCs and OVX–Obs *in vitro* (Supplementary Figs 19 and 20), we investigated whether long-term local delivery of miR-26a could rescue the attenuated bone-healing capacity under osteoporotic conditions. A critical-sized calvarial bone defect in an OVX-operated mouse was filled with a cell-free, miR-26a-releasing scaffold. After 8 weeks of implantation, there was minimal bone regeneration in all NC delivery groups, some bone regeneration at the periphery of the defect in the bolus and short-term miR-26a delivery groups, and complete bone-defect repair in the long-term miR-26a-delivery group (Fig. 7a–c). Consistently, bone mineral density (BMD) of newly formed bone in the long-term miR-26a delivery group was significantly higher than that in either bolus or short-term miR-26a delivery groups (Fig. 7d). The anabolic parameters, the ratio of Ob surface to bone surface (Ob.S/BS) and Ob number per bone perimeter (Ob.N/B.Pm), were found to be higher in the long-term miR-26a delivery group than in the short-term or bolus miR-26a delivery groups (Fig. 7e). OCN expression was significantly upregulated in the long-term miR-26a delivery group (Fig. 7f), suggesting that longer-term miR-26a delivery enhanced Ob activity. Bone-formation fronts were sequentially labelled using calcein and xylenol 7 days apart. There were intensive calcein and xylenol bands, and a larger width between two adjacent bands in mice with the long-term miR-26a delivery than those with short-term or bolus miR-26a delivery. The mineral apposition rate (MAR) and bone-formation rate in the long-term miR-26a delivery group were about two times higher than those in the short-term and bolus miR-26a delivery groups (Fig. 7g).

With tartrate-resistant acid phosphatase assay (Supplementary Fig. 21), the two catabolic parameters, the ratio of Oc.S/BS and the Oc.N/B.Pm, were found to be similar among the six groups (Fig. 7h), suggesting that osteoclasts were not the target of miR-26a.

### miR-26a directly targets *Gsk-3β*

To gain further insight into the mechanisms by which miR-26a regulates Ob activity, miRBase (the miRNA data base at http://www.mirbase.org) was used to predict the potential targets of miR-26a. Among the candidate target genes, glycogen synthase kinase-3β (Gsk-3β) has a miR-26a-binding site in its 3′ untranslated region (UTR). Inhibiting *GSK-3*β is known to improve bone mass and to significantly increase MAR and BMD in ovariectimized mice^35^,^36^.

**Figure 8:**
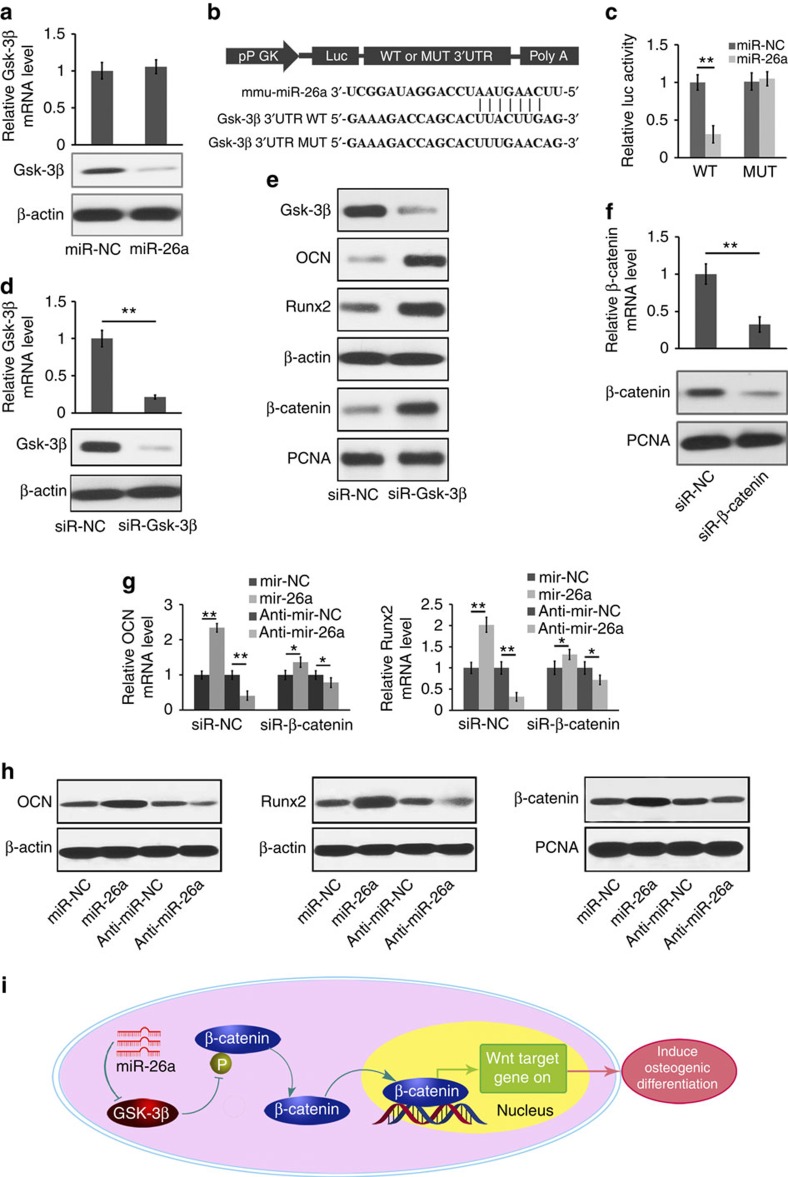
miR-26a targets Gsk-3β to functionally enhance osteoblast activity. (**a**) The effect of miR-26a, its NC (miR-NC) on Gsk-3β mRNA expression (top) and Gsk-3β protein level (bottom) in mouse primary osteoblast cells. (**b**) Schematic diagram illustrating the design of luciferase reporters with the WT Gsk-3β 3′UTR (Gsk-3β-3′UTR WT) or the site-directed mutant Gsk-3β 3′UTR (Gsk-3β-3′UTR mutated (MUT)). (**c**) The effect of miR-NC, miR-26a on luciferase activity in osteoblast cells transfected with either the Gsk-3β-3′UTR WT or the Gsk-3β-3′UTR MUT. (**d**) The Gsk-3β mRNA expression (top) and the amounts of Gsk-3β protein (bottom) in mouse primary osteoblasts after silencing Gsk-3β with a Gsk-3β-specific siRNA (siR-Gsk-3β). (**e**) The western blot analysis of Runx2, OCN and β-catenin protein in mouse primary osteoblasts after treatment with siR-Gsk-3β or its NC (siR-NC) in osteogenic medium for 7 days. (**f**) The β-catenin mRNA expression (top) and the β-catenin protein content (bottom) in mouse primary osteoblasts after silencing β-catenin with a β-catenin-specific siRNA (siR-β-catenin). (**g**) Real-time RT–PCR analysis of *Runx2* and *OCN* mRNA levels in mouse primary osteoblast cells after silencing β-catenin with siR-β-catenin under the treatment of miR-26a or anti-miR-26a or their corresponding NCs. (**h**) The western blot analysis of Runx2, OCN and β-catenin protein contents in mouse primary osteoblasts after treatment with miR-26a or anti-miR-26a or their corresponding NCs. (**i**) Schematic diagram of miR-26a-mediated osteoblast activity by modulating Gsk-3β/β-catenin signalling pathway in osteoblasts. Intracellular Gsk-3β level was downregulated by miR-26a. Subsequently, β-catenin phosphorylation by Gsk-3β was repressed in response to reduced Gsk-3β expression, and therefore the stabilization, accumulation and translocation of β-catenin from the cytoplasm to the nucleus to promote the transcription, which resulted in the induction of osteogenic differentiation. *P<0.05; **P<0.01. All experiments were performed in triplicate. *n*=5 per group. Data are mean±s.d. The full scans of blots in this figure are provided in Supplementary Fig. 24.

To test whether Gsk-3β has a binding site for miR-26a, the effects of miR-26a or its NC (miR-NC) on *Gsk-3β* mRNA and protein expression levels in mouse primary MSCs and Obs were examined. The amount of Gsk-3β protein in Obs was downregulated by miR-26a, but the *Gsk-3β* mRNA was not changed by miR-26a (Fig. 8a). To test whether miR-26a directly targets Gsk-3β, we constructed luciferase reporters that had either a wild-type (WT) Gsk-3β 3′UTR or a mutated Gsk-3β 3′UTR sequence of the miR-26a-binding site (Fig. 8b). It was found that miR-26a substantially inhibited the luciferase reporter activity of the WT Gsk-3β-3′UTR, but not that of the mutated Gsk-3β 3′UTR (Fig. 8c). To investigate the role of Gsk-3β in regulating osteoblastic activity, Gsk-3β-specific siRNA (siR-Gsk-3β) was used to silence Gsk-3β expression in both mouse primary MSCs and Obs. The intracellular Gsk-3β levels were substantially downregulated by siR-Gsk-3β compared with its NC (siR-NC; Fig. 8d). In contrast, Runx2 and OCN protein levels were significantly upregulated in response to the reduced Gsk-3β expression (Fig. 8e). To determine whether miR-26a functionally targets Gsk-3β in regulating Ob activity, β-catenin expression was knocked down using siRNA (Fig. 8f). The differences in *Runx2* and *OCN* mRNA levels under the treatment of miR-26a, anti-miR-26a or the NC were significantly reduced by the reduced β-catenin expression (Fig. 8g). Consistent with the mRNA expression changes, the protein expression levels of Runx2, OCN and β-catenin were enhanced by the miR-26a overexpression and reduced by the miR-26a inhibition (Fig. 8h). Mechanistically, we found that β-catenin phosphorylation by Gsk-3β was subsequently inhibited by siR-Gsk-3β treatment, leading to the stabilization, accumulation and translocation of β-catenin from the cytoplasm to the nucleus to promote transcription, which is known to induce osteogenic differentiation (Fig. 8i)^37^,^38^. Taken together, these results demonstrate that miR-26a upregulates Ob activity through functionally targeting Gsk-3β.

## Discussion

Systemic growth factor administration has been investigated for tissue regeneration, but frequently fail in clinical trials because of their short half-life *in vivo*^39^. Recently, miRNAs have emerged as novel therapeutic agents because of several significant advantages including the small size and a sequence that is often completely conserved among species^40^. A subset of miRNAs has shown therapeutic promise and is being actively pursued as clinical candidates for various disease indications, such as cancer, metabolic disease, fibrosis and inflammatory diseases among others^18^,^40^,^41^,^42^,^43^. In order for miRNAs to become successful therapeutic agents, many challenges remain to be overcome, including their efficient delivery, biological stability, long-term presentation and targeting^44^.

In this work, a non-viral HP vector with very high miRNA-binding affinity and negligible cytotoxicity has been developed. To synthesize an HP co-polymer, a low molecular weight PEI (weight average molecular weight: 800 Da) was first attached with a nitrile end group to form PEI-yne (Supplementary Fig. 1). Next, the hydroxyl end groups on a hyperbranched aliphatic polyester were converted into carboxyl end groups, which were utilized to conjugate PEG chains and azide groups (Fig. 2). The prepared PEI-yne was then attached on the modified HP utilizing the azide groups via click chemistry to form the designed final HP, containing both low molecular weight PEI and hydrophilic PEG chains (Fig. 2 and Supplementary Fig. 2). Since polyesters are unstable and degrade if reacted with the amino compound PEI^45^, the designed synthesis route using mild reaction conditions via click chemistry was critical for accomplishing the conjugation of cationic polymer PEI to the polyesters. For comparison, a group of LPs were synthesized via a combination of ring-opening polymerization and click chemistry (Supplementary Fig. 3).

Core-multishell architecture reported by others^46^,^47^ is based on a hyperbranched polymeric core grafted with different polymer chains in a sequential manner to form the multilayered shells; therefore, the concentric multilayered shells are part of the co-polymer. In the HP co-polymers that were developed in this work, two different hydrophilic polymers (PEG and PEI) were grafted on the hydrophobic core (Hx) simultaneously to form the multishell molecular structure (level-1). When the HP co-polymer and miRNA were complexed, the core-shelled HP co-polymer molecules further self-assembled into a larger nano-sized spherical aggregate (level-2) as illustrated in Fig. 3a (HP—middle column) and observed under TEM (Fig. 3b, HP—right panel). Here the ‘two levels of shells’ are not concentric. The level-1 molecular micelles are assembled into a spherical middle shell (Hx–PEI/miRNA) of a level-2 nano-sized sphere (the miRNA-loaded HP polyplex). We called this two-level distribution of miRNA as a ‘double-shell’ distribution. We hypothesized that such ‘double-shell’ charge distribution lowers toxicity (charge density) and increases charge accessibility to miRNAs and interaction area with cell membrane, serving as an advantageous miRNA vector, which is supported by the experimental results. To maintain a similar PEG/Hx ratio, the PEG chains have to be shorter for a higher branched Hx. The short PEG chains likely improve the charge (PEI) accessibility to miRNAs (thinner stealth layer) and the larger polyplex size likely increases the interaction area with cell membrane, thus increasing the cellular uptake of this polymeric vector.

Polyplexes can protect miRNAs from extracellular degradation on the journey into cells^48^. After the polymer/miRNA polyplexes cross the cell membrane and are enwrapped in endosomes, the cationic PEI component can buffer the acidic environment in the endosomal vesicles, preserving the biological activity of the miRNAs and causing endosomal swelling and lysis^26^, resulting in endosomal escape of the polyplexes. The polymer degrades in the cytosol and releases miRNA from the polyplexes. These polyplexes are very stable even under rigorous sonication and, therefore, can be stably encapsulated into biodegradable PLGA MS to achieve controllable release of bioactive miRNAs for the desired long durations. In addition, the PLGA MS incorporating HP/miR-26a polyplexes are attached on NF PLLA scaffolds, where the released HP/miRNA polyplexes locally transfect endogenous cells to regenerate a critical-sized bone defect without externally introducing cells. PLLA and PLGA are known to release acid during degradation. For polymer scaffolds with high porosity as used in this study (∼95%), the acid release (pH variation) is not of a significant concern^11^. Furthermore, this 3D scaffold-defined two-stage delivery strategy can circumvent uncontrollable off-target effects in other delivery approaches. The dynamic labelling of bone-formation front reveals that the calvarial bone regeneration starts from the bottom, suggesting that the transfected bone-forming stem cells are mainly recruited from the dura.

With multiple functions, miR-26a was reported to regulate angiogenesis by targeting BMP/SMAD1 signalling in endothelial cells^49^ and to regulate osteoclast formation *in vitro* by suppressing connective tissue growth factor (CTGF)^50^. Luzi *el al.* showed that miR-26a gene was upregulated during osteogenic differentiation of adipose-derived MSCs, but inhibition of miR-26a upregulated a few osteogenic genes of the same cells by targeting the *SMAD1* gene^51^. However, this study was conducted on a different stem cell type (adipose-derived), at the gene expression level, and using cell cultures only. Li found that miR-26a enhanced osteogenic differentiation of bone marrow-derived MSCs^47^, but neither identified the target gene nor regenerated bone without externally introduced cells when a state-of-the-art commercial gel was used to deliver this miRNA. In this work, we demonstrated that the 3D two-stage miR-26a delivery enhanced multiple osteogenic genes both *in vitro* and *in vivo*, resulting in repair of the critical-sized calvarial bone defects without adding cells.

Mechanistically, we find that osteogenic action of miR-26a is through functionally targeting Gsk-3β to increase the Ob activity rather than to suppress osteoclast activity. In addition, the data show that the long-term (longer than 1 month) highly efficient miR-26a delivery is required to achieve the elevated expression of multiple osteogenic genes for a long duration to fully harness the osteogenic capacity of the host cells. In addition, we investigated the possibility of miR-26a stimulating the osteocyte differentiation of MSCs, but found that miR-26a overexpression had no statistically significant effect on the expression levels of two osteocyte markers (DMP1 and SOST) at either gene or protein levels (Supplementary Fig. 22). Furthermore, we treated primary osteocytes (isolated following a published protocol^52^) with miR-26a. Osteocytes are known to function as mechanosensors that modulate the activity of Obs to form new bone and to regulate osteoclasts to resorb old bone through producing sclerostin, an inhibitor of Wnt signalling^53^. A recent study found that absence of DMP1 resulted in defective osteocyte maturation, leading to pathological changes in bone mineralization^54^. Therefore, we also examined the effect of miR-26a on the SOST and DMP1 expression levels in osteocytes, and found that miR-26a could effectively transfect Obs and osteocytes with the HP3 vector (Supplementary Fig. 23a,b) but did not have statistically significant effect on the two markers of osteocytes (Supplementary Fig. 23c). Therefore, we concluded that 3D two-stage miRNA delivery could transfect all the osteoblastic lineage cells (MSCs, Obs and osteocytes), but functionally primarily acted on MSCs and Obs, without substantially affecting the activity of osteocytes.

In tissue engineering, the scaffold, cells and biological factors play roles in accelerating or improving the quality of regeneration. The use of a growth factor^29^,^55^ and the design of an advanced scaffold composition^56^ or pore/surface structure^31^,^33^,^57^,^58^,^59^ have both been demonstrated to facilitate bone formation or/and improve the quality of bone repair. However, to achieve robust high-quality and large-sized bone regeneration as required in clinic, the utilization of more than one single material or biological factor is likely needed. In this work, we developed the novel 3D scaffold-defined controlled miRNA delivery technology, which enabled the prolonged expression of multiple osteogenic genes at their therapeutic levels, leading to repair of critical-sized bone defect without adding cells.

Approximately 200 million people suffer from osteoporosis worldwide^60^, and their bone-healing capacity is severely compromised. In this work, the long-term highly efficient miR-26a delivery is shown to locally rescue the osteogenic capacity of osteoporosis-impaired osteogenic cells for bone regeneration, demonstrating the potential to repair bone for osteoporotic patients and possibly patients with other impaired bone regeneration capacities. This technology could deliver other therapeutic nucleic acids (DNAs, mRNAs, siRNAs, miRNAs and so on) or their inhibitors to regenerate other tissues or to treat other diseases.

## Methods

### Materials

PEI (molecular weight (Mw) 800 Da and 25 kDa), PEG methyl ether (Mw 2,000, 5,000, 10,000 and 20,000 Da) and hyperbranched 2,2-bis(hydroxymethyl)propionic acid (bis-MPA) polyester (H20: 16 hydroxyl, H30: 32 hydroxyl and H40: 64 hydroxyl) were purchased from Sigma-Aldrich (USA) and were used as received. The fluorescent analogue, NBD cholesterol, a probe that localizes in the membrane’s interior and is useful for investigating lipid transport processes as well as lipid–protein interactions was purchased from Life Technologies. α-Azido-ε-caprolactone (α-N_3_-ε-CL) was synthesized as described in the literature^61^.

*Synthesis of PEI-yne*. 1,1′-Carbonyldiimidazole (7.30 g, 45 mmol) was dissolved in 50 ml of dichloromethane. Propargyl alcohol (1.50 ml, 26 mmol) was added dropwise. The reaction mixture was stirred at room temperature for 1 h and washed using 40 ml of water three times. The organic phase was dried with anhydrous magnesium sulfate and the filtrate was concentrated under reduced pressure to obtain the propargyl ester of carbonylimidazole as a white solid. Yield: 89%. ^1^H NMR (400 MHz, CDCl_3_): *δ* 8.17 (s, 1H, NC*H*N), 7.46 (s, 1H, NC*H*CHN), 7.10 (s, 1H, NCHC*H*N), 5.01 (s, 2H, OC*H*_2_C), 2.65 (s, 1H, C≡C*H*).

PEI (Mw 800 Da, 2.40 g, 3.0 mmol) was dissolved in a mixture of chloroform (25 ml) and methanol (5 ml). A solution of propargyl ester of carbonylimidazole (0.45 g, 3.0 mmol) in 4.5 ml of chloroform was added dropwise over a period of 30 min in an ice/water bath. After the mixture was refluxed for 6 h, the solvents were removed by rotary evaporation. The residue was dissolved in 10 ml of water. Then, the solution was neutralized with 1 N hydrochloric acid standard solution and washed with 5 ml of chloroform three times. The aqueous phase was lyophilized to obtain PEI-yne as a yellow solid. Yield: 85%. FT-IR (KBr): 2,130 cm^−1^ (*v*_C≡CH_), 1,716 cm^−1^ (*v*_CONH_), 1,591 cm^−1^ (*v*_CONH_), 1,466 cm^−1^ (*v*_C-N_). ^1^H NMR (400 MHz, D_2_O): *δ* 4.68 (s, 2H, OC*H*_2_C), 3.81–2.60 (m, 74H, NC*H*_2_), 2.47 (s, 1H, C≡C*H*).

*Synthesis of PEG-HP-N_3_*. Boltorn hyperbranched bis-MPA polyester Hx (H20: 16 hydroxyl, H30: 32 hydroxyl, H40: 64 hydroxyl; 2.00 g, *ca.* 17.5 mmol of hydroxyl) and triethylamine (0.5 ml, 3.5 mmol) were dissolved in 50 ml of tetrahydrofuran (THF). Succinic anhydride (3.50 g, 35 mmol, twofold molar excess) was added. The reaction mixture was stirred at room temperature for 24 h and floc-like solid precipitates were washed with THF and diethyl ether to remove the residual succinic anhydride. The residue was dried under vacuum to obtain Hx-COOH as a white solid. Yield: 62–73%. Fourier transform infrared (FT-IR; KBr): 2,978 cm^−1^ (*v*_O–H_), 2,940 cm^−1^ (*v*_O–H_), 1,740 cm^−1^ (*v*_C=O_), 1,400 cm^−1^ (*v*_C–O_), 1,245 cm^−1^ (*δ*_O–H_); ^1^H NMR (400 MHz, D_2_O): *δ* 4.29 (br s, C*H*_2_O), 2.84–2.51 (m, C*H*_2_C*H*_2_COOH), 1.27 (s, C*H*_3_).

Hx-COOH (3.2 mmol of carboxyl), N,N′-dicyclohexylcarbodiimide (0.990 g, 4.8 mmol) and 4-dimethylaminopyridine (0.586 g, 4.8 mmol) were dissolved in 50 ml dimethylformamide. 2,2-bis(azidomethyl)propane-1,3-diol (0.300 g, 1.6 mmol) and PEG (Mw 2,000 Da, 3.200 g, 1.6 mmol) were added. The reaction was stirred at room temperature for 24 h. The reaction mixture was filtered and concentrated. The residue was then transferred into a dialysis bag (molecular weight cut-off (MWCO) 3,500 Da, Fisherbrand regenerated cellulose) and dialysed against a continuous flow of deionized water for 2 days, during which the water was renewed every 8 h. A flocculent solid product was obtained by lyophilization. Yield: 34–46%. FT-IR (KBr): 3,433 cm^−1^ (*v*_O–H_), 2,105 cm^−1^ (*v*_N3_), 1,742 cm^−1^ (*v*_C=O_), 1,342 cm^−1^ (*δ*_O–H_), 1,147 cm^−1^ (*v*_C–O_), 1,108 cm^−1^ (*v*_C–O–C_). ^1^H NMR (400 MHz, CDCl_3_): *δ* 4.22 (br s, C*H*_2_O of Hx), 4.06 (br s, CC*H*_2_OH and CC*H*_2_OOC), 3.88–3.56 (m, OC*H*_2_C*H*_2_ of PEG), 3.46 (br s, C*H*_2_N_3_), 3.38 (s, C*H*_3_ of PEG), 2.78–2.56 (m, C*H*_2_C*H*_2_COO of Hx), 1.25 (s, C*H*_3_ of Hx).

*Synthesis of PEG–HP–PEI via click chemistry*. PEG-HP-N_3_ (1 equiv. N_3_), PEI-yne (2 equiv. alkynyl), copper(I) bromide (2 equiv.) and triethylamine (2 equiv.) were dissolved in THF–water mixture (1:1 by volume). The mixture was stirred for 3 h at 35 °C. The solution was then transferred into a dialysis bag (MWCO 3,500 Da) and dialysed against a continuous flow of deionized water for 2 days, during which the water was renewed every 8 h. A flocculent solid product was obtained by lyophilization. Yield: 44–60%. FT-IR (KBr): 3,435 cm^−1^ (*v*_O–H_), 2,106 cm^−1^ (*v*_N3_), 1,743 cm^−1^ (*v*_C=O_), 1,635 cm^−1^ (*v*_triazole_), 1,343 cm^−1^ (*δ*_O–H_), 1,150 cm^−1^ (*v*_C–O_), 1,113 cm^−1^ (*v*_C–O–C_); ^1^H NMR (400 MHz, DMSO (dimethylsulphoxide)-*d*_6_): δ 4.15 (br s, C*H*_2_O of Hx), 3.99 (br s, CC*H*_2_OH and CC*H*_2_OOC), 3.73–3.46 (m, OC*H*_2_C*H*_2_ of PEG), 2.72–2.54 (m, C*H*_2_C*H*_2_COO of Hx), 1.18 (s, C*H*_3_ of Hx).

*Linear PEG-P(N_3_CL-CL) polymer synthesis*. A round-bottom flask pre-treated with trimethylchlorosilane was charged with 1.0 g of PEG (Mw 5,000, 10,000 or 20,000 Da) and heated at 100 °C under reduced pressure (oil pump) for 4 h. α-N_3_-ε-CL (0.33 g, 2.0 mmol), ε-caprolactone (ε-CL, 0.456 g, 4.0 mmol) and 60 μl of 0.1 M Sn(Oct)_2_ solution in anhydrous toluene were added. The flask was evacuated and charged with nitrogen three times, and then sealed under vacuum with a magnetic stirring bar inside. After the mixture was stirred at 130 °C for 24 h, the polymerization was quenched by immersing the flask in an ice/water bath. The product was purified by precipitation from chloroform with cold methanol-diethyl ether mixture (1:1 by volume) three times and dried under vacuum to give a white solid, PEG-P(N_3_CL-CL) (PEG–LP). Yield: 84–88%. FT-IR (KBr): 2,111 cm^−1^ (*v*_N3_), 1,749 cm^−1^ (*v*_C=O_), 1,149 cm^−1^ (*v*_C–O_), 1,113 cm^−1^ (*v*_C–O–C_). ^1^H NMR (400 MHz, CDCl_3_): *δ* 4.22–4.11 (t, OCOCH(N_3_)CH_2_CH_2_CH_2_C*H*_2_), 4.08–3.96 (t, OCOCH_2_CH_2_CH_2_CH_2_C*H*_2_), 3.88–3.80 (t, OCOC*H*(N_3_)CH_2_CH_2_CH_2_CH_2_), 3.74–3.58 (br s, OC*H*_2_C*H*_2_ of PEG), 3.38 (s, C*H*_3_ of PEG), 2.38–2.22 (t, OCOC*H*_2_CH_2_CH_2_CH_2_CH_2_), 1.83–1.58 (m, C*H*_2_CH_2_C*H*_2_CH_2_O of N_3_CL and CL), 1.45–1.28 (m, CH_2_C*H*_2_CH_2_CH_2_O of N_3_CL and CL).

*Synthesis of PEG–LP–PEI via click chemistry*. PEG-P(N_3_CL-CL) (0.16 mmol of N_3_), PEI-yne (0.162 g, 0.55 mmol of alkynyl), copper(I) bromide (0.066 g, 0.46 mmol) and triethylamine (0.047 g, 0.46 mmol) were dissolved in a THF–water mixture (1:1 by volume). The mixture was stirred for 3 h at 35 °C. The solution was then transferred into a dialysis bag (MWCO 3,500 Da, Fisherbrand regenerated cellulose) and dialysed against a continuous flow of deionized water for 2 days, during which the water was renewed every 8 h. The flocculent solid product PEG-P(N_3_CL-CL)–PEI (PEG–LP-PEI) was obtained by lyophilization. Yield: 68–75%. FT-IR (KBr): 2,109 cm^−1^ (*v*_N3_), 1,750 cm^−1^ (*v*_C=O_), 1,637 cm^−1^ (*v*_triazole_), 1,148 cm^−1^ (*v*_C–O_), 1,113 cm^−1^ (*v*_C–O–C_). ^1^H NMR (400 MHz, DMSO-*d*_6_): *δ* 4.14–4.03 (m, OCOCH_2_CH_2_CH_2_CH_2_C*H*_2_), 3.62 (br s, OC*H*_2_C*H*_2_ of PEG), 2.35–2.20 (t, OCOC*H*_2_CH_2_CH_2_CH_2_CH_2_), 1.84–1.56 (m, C*H*_2_CH_2_C*H*_2_CH_2_O of N_3_CL and CL), 1.47–1.30 (m, CH_2_C*H*_2_CH_2_CH_2_O of N_3_CL and CL).

### Polymer characterization

The FT-IR spectra were recorded on a Perkin Elmer BX spectrometer. ^1^H NMR analyses were performed on a Varian INOVA-400 spectrometer operating at room temperature. Molecular weight and molecular weight distribution were determined with a Waters 440 gel permeation chromatograph at 35 °C. THF was used as an eluent at a flow rate of 1.0 ml min^−1^. Polystyrene standards with narrow distributions were used to generate a calibration curve. Elemental analyses were carried out by Atlantic Microlab Inc. The molecular weights of polymers were also carried out using MALDI-TOF mass spectrometer with a Bruker Autoflex Speed run in the linear mode. The polymer was dissolved in water (1 mg ml^−1^). 2,5-Dihydroxybenzoic acid (purchased from Sigma-Aldrich) was used as a matrix and 50/50 acetonitrile/water with 0.1% trifluoroacetic acid as a solvent. Three microlitres of each sample solution was mixed with three microlitres of matrix solution, and the mixture of matrix and sample was spotted on the target plate and evaporation-dried. The mass spectrometer was calibrated with the protein cytochrome-*C* in a sinapinic acid matrix.

### Cytotoxicity assay of polymer

The cytotoxicity assay was carried out using Obs and an MTT assay. The Obs were seeded in 96-well plates at an initial density of 5,000 cells per well in 200 μl of DMEM complete medium. The cells were allowed to grow for 24 h. The original medium was replaced with 100 μl of fresh medium. PEG–PE–PEI, PEG–Hx–PEI, PEI 800 Da or PEI 25 kDa solution was added to the medium at 10 μg ml^−1^ concentration of nitrogen in medium. Each polymer was replicated in four wells. Treated cells were incubated at 37 °C under a humidified atmosphere of 95% air and 5% CO_2_ for 24 h. MTT reagent (20 μl in PBS, 5 mg ml^−1^) was added to each well, and the cells were incubated for another 4 h at 37 °C. DMSO (100 μl) was added to each well until all crystals dissolved. The absorbance at 570 nm of the solution in each well was recorded using a ThermoElectron 3001 Varioskan Flash Spectral Scanning Microplate Reader. Cell viability was calculated according to the following equation: cell viability (%)=(optical density (OD)_sample_−OD_blank_)/(OD_control_−OD_blank_) × 100, where OD_sample_ is the absorbance of the solution of the cells cultured with the polymer or PEI; OD_blank_ is the absorbance of the medium; and OD_control_ is the absorbance of the solution of the cells cultured with the medium only.

### Preparation of polyplexes and agarose gel retardation

Desired polymer solutions (1.0 mg ml^−1^) were added slowly to RNA solutions containing 60 pmol of RNA. The amount of polymer added was calculated on the basis of chosen N/P ratios of polymer/RNA (nitrogen atoms of the polymer over phosphates of RNA). The mixture was incubated at room temperature for 30 min for complex formation. Polyplex solution (10 μl) mixed with 4 μl of 1 × loading buffer was loaded on a 0.7% agarose gel containing GelRed with Tris-acetate (TAE) running buffer (pH 8) and was electrophoresed at 80 V for 45 min. RNA bands were visualized with an ultraviolet (254 nm) illuminator and photographed with a BioSpectrum Imaging System (USA).

### TEM study of the morphology of polymer/miRNA polyplexes

A novel negative staining method for TEM was established to visualize the miRNA in this work. W-incorporated miRNA was prepared via activated tungstic acid to observe the miRNA under TEM. Briefly, 10 ml of sodium tungstate aqueous solution (0.15 mol l^−1^) was added dropwise to 6 ml of HCl aqueous solution (0.8 mol l^−1^) at room temperature. The precipitate was collected and washed to obtain the activated tungstic acid. A desired quantity of activated tungstic acid was added to an aqueous miRNA solution with a W/P molar ratio of 3. The W-incorporated miRNA solution was purified by centrifugation and stored at −80 °C.

For the TEM study, the W-incorporated miRNA solution was used to prepare polymer/miRNA polyplexes. The polyplexes at an N/P ratio of 10 were prepared by adding an appropriate volume of W-incorporated miRNA solution to an appropriate volume of polymer solution (1.0 mg ml^−1^) and was incubated at room temperature for 30 min. One drop of polymer/miRNA polyplex solution was added on a carbon-coated copper grid. The grid was allowed to dry under ambient conditions. A JEOL JEM-3011 TEM was used to characterize the morphology of polymer/miRNA polyplexes.

### Particle size and zeta potential measurements

The particle size and zeta potential were measured using a Beckman Coulter DelsaNano C Submicron Particle Size Analyzer at room temperature. The polyplexes at a N/P ratio of 10 were prepared by adding an appropriate volume of RNA solution to an appropriate volume of polymer solution (1.0 mg ml^−1^) and incubated at room temperature for 30 min. The polyplexes were then diluted with RNase-free water to 1.0 ml volume before measure.

### miRNA releasing PLGA MS and MS scaffolds

PLGA MS containing polymer/miRNA polyplexes were fabricated using a modified water-in-oil-in-water (w/o/w) double emulsion method^28^. Briefly, 30 mg of PLGA was dissolved in 1 ml of dichloromethane. Hundred microlitres of polymer/miRNA polyplex aqueous solution with a N/P of 10 containing 1.8 nmol of miRNA was added into the above solution and emulsified with a probe sonicator at 50 W (Virsonic 100, Gardiner, NY). This primary w/o emulsion was then emulsified into 10 ml of polyvinyl alcohol (PVA) solution (1% wt/vol) under sonication at 90 W to form the w/o/w emulsion. The resulting double emulsion was magnetically stirred at room temperature for 12 h to evaporate the solvent. The PLGA MS were collected by centrifugation and washed three times with water and freeze-dried.

The PLLA NF scaffolds 5 mm in diameter and 2 mm in thickness were prefabricated by a combination of phase separation and sugar leaching techniques previously described^62^. The PLGA MS were incorporated on PLLA NF scaffolds using a post-seeding method^29^. Briefly, PLGA MS were dispersed in hexane with 0.1% span 80 and were seeded on NF scaffolds dropwise. Then, the scaffolds were subjected to vapour of a mixed solvent of hexane/THF (9:1 by volume) for 30 min, and were subsequently dried under vacuum for 3 days to remove the solvents.

### Characterization of PLGA MS and MS scaffolds

The morphology and size of the scaffolds before and after MS incorporation were characterized using scanning electron microscopy (Philips XL30 FEG SEM). The scaffolds were coated with gold using a sputter coater (DeskII, Denton vacuum Inc.). During the process of gold-coating, the gas pressure was 50 mtorr and the current was 40 mA. The coating time was 120 s. Samples were analysed at 15 kV.

The miRNA-loading content and encapsulation efficiency were determined using ultraviolet spectrophotometric analysis. First, 1.0 mg of PLGA MS containing polymer/miRNA polyplex was dissolved in 1.0 ml of dichloromethane. Then, 0.5 ml of RNase-free water was added to extract miRNA. The extraction process was repeated several times. The miRNA concentration was measured using a ThermoElectron 3001 Varioskan Flash Spectral Scanning Microplate Reader.

### *In vitro* miRNA release study

The release profiles of miRNA-loaded PLGA MS scaffolds were examined in PBS (pH 7.4, 0.1 M). The scaffolds were placed in 0.2 ml PBS and shaken at 50 r.p.m. at 37 °C. At predetermined time intervals, the release medium was withdrawn and replaced with pre-warmed fresh PBS. QuantiFluor RNA Dye working solution (100 μl) was added to each well in a 96-well plate containing 100 μl of unknown, blank or standard sample, and mixed briefly. After being incubated for 5 min at room temperature (protected from light), the miRNA concentration of the sample was measured using a ThermoElectron 3001 Varioskan Flash Spectral Scanning Microplate Reader.

### Primary cell culture

The primary mouse Obs were isolated from adult mouse calvaria following a standard protocol^63^. Briefly, the parietal and frontal calvarial bones were dissected under a dissecting microscope and subjected to sequential digestion. The bone pieces were treated with 2 mg ml^−1^ collagenase II (Gibco, USA) for 2 × 30 min at 37 °C in a shaking water bath, and then transferred to 0.25% trypsin solution (Gibco) for 30 min and subsequently treated with collagenase II (Gibco) for 2 × 30 min. Osteoblasts were obtained after the above five digestions and fractionations, and then combined and cultured in DMEM supplemented with 10% fetal bovine serum (FBS; Gibco) at 37 °C in a humidified atmosphere of 5% CO_2_ in air.

MSCs were isolated from 4- to 6-week-old female C57BL/6J mice (Charles River Laboratories, Wilmington, MA) as described by others^19^. Briefly, all soft tissues were removed from hind legs and the bones were serially washed with PBS. Both ends of the bone were cut and marrow was flushed with complete culture medium through the cavity using a syringe. Cells were cultured in a medium containing 20% FBS at 37 °C in a humidified atmosphere of 5% CO_2_ in air. After 5–7 days, nonadherent cells were removed by changing the medium and replacing it with fresh complete medium. Cells from passages 3–5 were used in all experiments.

To induce osteogenic differentiation, cells were cultured in an osteogenic medium containing 50 μg ml^−1^ ascorbic acid, 10 mM β-glycerophosphate and 10 nM dexamethasone. After induction for 2 weeks, cells were fixed with 70% ethanol and stained with 2% Alizarin red (Sigma-Aldrich) according to the manufacturer’s protocol to detect mineral deposition.

### OVX-induced osteoporotic mouse model

The female C57BL/6J mice used were maintained under standard animal housing conditions (12-h light, 12-h dark cycles and free access to food and water). The mice were ovariectomized or sham-operated at 3 months of age. At 2 months after surgery (5 months of age), the proximal tibia from ovariectomized and sham-operated mice were collected for microCT analysis. The animal protocol was approved by the University of Michigan Committee on Use and Care of Laboratory Animals.

### Cellular uptake of miRNA and miRNA expression in cells

For cellular uptake and miRNA expression analysis of miRNA/polymer complexes and lipoplexes *in vitro*, polymers and liposome were labelled following the standard protocols^64^. Briefly, FITC (2.0 mg) and polymer (16.0 mg) in methanol (2 ml) were stir-mixed overnight at room temperature. The solution was then transferred into a dialysis bag (MWCO 3,500 Da, Fisherbrand regenerated cellulose) and dialysed against a continuous flow of deionized water for 2 days, during which the water was renewed every 8 h. The flocculent FITC-polymer product was obtained by lyophilization. NBD cholesterol (10% by weight) was added to Lipofectamine 2000 solution to fluorescently label lipoplexes.

The *in vitro* transfection efficiency was evaluated using primary Obs. Obs were plated at a density of 4,000 cells per well on an eight-chambered coverglass (Nunc, USA) and incubated at 37 °C in a humidified atmosphere of 95% air and 5% CO_2_ until the cells reached ∼70% confluence. At the time of transfection, the medium in each well was replaced with 400 μl fresh DMEM medium. The FITC-labelled polymer or NBD cholesterol-labelled liposome combined with Cy3-labelled miRNA to form polyplexes or lipoplexes as described above and were subsequently incubated with cells at 37 °C. At 48 h post transfection, cells were observed using confocal laser scanning microscopy (Nikon C1-si TE2000, Japan) and RNAs were extracted for real-time reverse transcriptase PCR (RT–PCR) analysis.

To quantify the cellular uptake of miRNA-containing polyplexes or lipoplexes released *in vitro* from PLGA MS on the NF PLLA scaffolds, the polyplex- or lipoplex-containing PLGA MS were immobilized on the NF PLLA scaffolds, placed in 0.2 ml of PBS and shaken at 50 r.p.m. at 37 °C for 2 days. The release medium was collected and co-cultured with the Obs under the above-described conditions. At 48 h post transfection, fluorescent images of transfected cells were recorded, RNA was extracted and analysed using real-time RT–PCR.

To examine the cellular uptake of Cy3-labelled polyplexes and Cy3-labelled agomir *in vivo*, the NF PLLA scaffolds with immobilized PLGA MS containing FITC-labelled polyplexes or Cy3-labelled agomir were implanted in subcutaneous pockets on the back of nude mice. At 2 weeks post surgery, the implants were harvested for fluorescent imaging, RNA extraction and real-time RT–PCR analysis.

To quantify the amount of polyplexes, lipoplexes or agomir taken up by cells, the captured fluorescence images of labelled miRNA, polyplexes or lipoplexes were analysed using the Image-pro plus 6.0 image analysis software. The fluorescence intensity was measured and compared with the area expressing 4,6-diamidino-2-phenylindole (DAPI)-derived fluorescence in nuclei.

### Subcutaneous implantation

After the controlled miRNA release scaffold system was established, scaffolds immobilized with 64 or 6.5 k PLGA MS containing miR-26a/polymer, NC/polymer polyplexes or with blank MS were seeded with 1 × 10^5^ mouse mesenchymal stem cells and implanted in subcutaneous pockets on the back of nude mice as described in ref. 65. Briefly, 6–8-week-old male nude mice (Charles River Laboratories) were anaesthetized with 2% inhalation of isofluorane. Two dorsal midsagittal incisions were made on the disinfected back. One subcutaneous pocket was created on each side of each incision using blunt dissection, and one scaffold was implanted into each pocket. Four samples were implanted randomly for each group (*n*=5). For transfection efficiency and miRNA expression detection, mice were killed and the implants were harvested at 2 weeks. For bone-formation examination, the implants were harvested at 8 weeks for subsequent microCT analysis. The animal procedure was approved by the University of Michigan Committee on Use and Care of Laboratory Animals.

### Calvarial bone-defect model construction

C57BL/6J mice were divided into three groups randomly. Animals were anaesthetized with 2% inhalation of isofluorane. A linear scalp incision was made from the nasal bone to the occiput, and full-thickness flaps were elevated. The periosteum overlying the calvarial bone was completely resected. A trephine was used to create a 5-mm craniotomy defect centred on the parietal calvarial bone, and the wounds were copiously irrigated with Hanks’ balanced salt solution while drilling. The calvarial disk was removed carefully to avoid injury to the underlying dura or brain. After careful haemostasis, the cell-free miRNA-releasing scaffolds were placed in the defects. The incisions were closed with 4-0 braided absorbable sutures (Covidien, USA), and the mice recovered from anaesthesia on a heating pad. All mice were killed and calvaria were harvested at 8 weeks after the implantation.

### Micro CT analysis

For the proximal tibia analysis, specimens were embedded in 1% agarose and placed in a 19-mm tube and scanned over the entire length of the tibia using a microCT system (μCT100 Scanco Medical, Basserdorf, Switzerland). Scan settings were as follows: voxel size 15 mm, 70 kVp, 114 mA, 0.5 mm AL filter and integration time 500 ms. Analysis was performed using the manufacturer’s evaluation software, and a fixed global threshold of 28% (280 on a greyscale of 0–1,000) for cortical bone and 18% for trabecular bone was used to segment bone from non-bone areas. A 0.75-mm region of trabecular bone was analysed immediately below the growth plate, and a 0.5-mm cortical region was analysed located 3 mm proximal of the tibiofibular joint. For the calvarial bone analysis, the region of the parietal calvarial bone was scanned with a fixed global threshold of 20% (200 on a greyscale of 0–1,000). All the trabecular bones from each selected slice were segmented for 3D reconstruction to calculate the following parameters: BMD and relative bone volume (BV/TV).

For calvarial bone-defect repair examination using microCT (Siemens AG, Germany), the specimens were fitted in a cylindrical sample holder with the coronal aspect of the calvarial bone in a horizontal position. Specimens were scanned with the scanning direction parallel to the coronal aspect of the calvarial bone with a fixed isotopic voxel size of 12 mm, preformed to cover the entire thickness of the calvarial bone. Analysis was performed using the manufacturer’s evaluation software and a fixed global threshold of 20% (200 on a greyscale of 0–1,000). A 5-mm round region of interest centred around the epicentre of the defect was analysed. On 3D images of the specimen, bone volume (mm^3^) and BMD in the defect site were measured directly.

### Bone histomorphometric analysis

For examination of bone formation, mice were injected sequentially with calcein and xylenol, with the injections 7 days apart, and killed 2 days after the final injection. The calvaria was fixed in ethanol and embedded without decalcification. Fifteen-micrometre sections in the calvarial bone defect were obtained using a Leica CM 1950 microtome (Leica Microsystems, Germany). The sections were evaluated with fluorescence microscopy using a confocal microscope (Eclipse C1 Plus, Nikon, Japan). For osteoclast detection, the sections were stained for tartrate-resistant acid phosphatase activity using a leukocyte acid phosphatase staining kit (Sigma-Aldrich). Bone static histomorphometric analyses for Ob.S/BS, Oc.S/BS, Ob.N/B.Pm and Oc.N/B.Pm, as well as bone dynamic histomorphometric analyses for MAR, bone-formation rate/BS, were performed using the Image J software (NIH, USA). Bone histomorphometric parameters were calculated and expressed according to the standardized nomenclature for bone histomorphometry.

### Bone histological analysis

The specimens (from subcutaneous implantation and calvarial bone-defect repair models) were fixed with 4% formalin, decalcified with 10% EDTA and subsequently embedded in paraffin. Coronal sections (6-μm thick) were stained with haematoxylin and eosin. For immunofluorescence analysis of OCN expression in newly formed tissue in the defect area, the slides were de-paraffinized and cooked in citrate buffer (2.1 M citric acid, pH 6.0) at 120 °C for 30 min for antigen retrieval. After blocking in serum, the sections were incubated overnight at 4 °C with a rabbit polyclonal primary antibody to OCN (1:100, Abcam). After three washes in PBS, the sections were incubated with FITC-conjugated donkey antibody to rabbit IgG (1:400, Santa Cruz) for 1 h. NC experiments were performed by omitting the primary antibody. The sections were mounted with the medium containing DAPI (Vector Laboratories) and then examined under a confocal microscope (Eclipse C1 Plus, Nikon).

### RNA extraction and real-time RT–PCR

The total miRNA from the collected cells was extracted using the miRNeasy Mini Kit (Qiagen, USA). Briefly, the cells were collected in a reaction tube, lysed with 700 μl QIAzol and mixed with 140 μl chloroform. After being centrifuged at 12,000*g* for 15 min at 4 °C, the upper aqueous phase was transferred to an RNeasy Mini spin column in a 2-ml collection tube and mixed with 100% ethanol. After being washed with 700 μl Buffer RWT and 500 μl Buffer RPE, the total miRNA was collected for real-time PCR analysis.

Total RNA from bone tissues or cells was extracted using the RNeasy Mini Kit (Qiagen). cDNAs were synthesized using a Geneamp PCR (Applied Biosystems) with TaqMan reverse transcription reagents and 10 min incubation at 25 °C, 30 min reverse transcription at 48 °C and 5 min inactivation at 95 °C. One microlitre cDNA was PCR-amplified using Platinum Taq DNA Polymerase (Invitrogen). Two microlitres of a 1:10 dilution of the synthesized cDNA was used for real-time PCR. Primers used are listed below^66^: *Runx2* (5′- CCGCACGACAACCGCACCAT -3′ and 5′- CGCTCCGGCCCACAAATCTC -3′); *BSP* (5′- GTCAACGGCACCAGCACCAA -3′ and 5′- GTAGCTGTATTCGTCCTCAT -3′); *OCN* (5′- CGGCCCTGAGTCTGACAAA -3′ and 5′- ACCTTATTGCCCTCCTGCCTT -3′); *β-actin* (5′- CAGGATTCCATACCCAAGAAG -3′ and 5′- AACCCTAAGGCCAACCGTG -3′); *GSK-3β* (5′- GGAACTCCAACAAGGGAGCA -3′ and 5′- TTCGGGGTCGGAAGACCTT -3′); *β-catenin* (5′- AAATCCAGGTGGACAATGG -3′ and 5′- CTAGAGCAGACAGATAGCACC -3′); *PCNA* (5′- GAGCAAGGAATCCCAGAACAGG -3′ and 5′- CCAAGCTCCCCACTCGCAGAAAAC -3′); *SOST* (5′- CCTTGCCTTGCCTGCCTGCTTGTA -3′ and 5′- GTCCGTTCGGGCGCCACCACTTC -3′); *Alp* (5′- GGGACTGGTACTCGGATAACGA -3′ and 5′- CTGATATGCGATGTCCTTGCA -3′); *DMP1* (5′- GAAGACTGTTATCCTCCTTACG -3′ and 5′- GTGATCCCCTTTAGATTCTTCC -3′).

### Western blot analysis

Total protein or nuclear protein was extracted using the EpiQuik whole-cell extraction kit or EpiQuik nuclear protein extraction kit (Epigentek, USA). The protein concentration was measured following the manufacturer’s instruction (Bio-Rad, USA). Protein was applied and separated on 4–15% NuPAGE gels (Bio-Rad) and transferred to polyvinylidene difluoride membranes (Millipore, USA). The membranes were blocked with 5% bovine serum albumin and incubated with specific antibodies (1:1,000 dilution) overnight. Horseradish peroxidase-conjugated IgG (1:10,000 dilution) from Santa Cruz Biotechnology (Santa Cruz, USA) was used to treat the membrane for 1 h, after which the membranes were enhanced with a SuperSignal West Pico Chemiluminescent Substrate (Thermo, USA). The relative amounts of the transferred proteins were quantified with scanning the autoradiographic films. Total protein or nuclear protein was normalized to the corresponding β-actin or PCNA level.

### Luciferase reporter assay

Gsk-3β mRNA 3′UTRs containing the miR-26a-binding sequences for the mouse Gsk-3β gene (gene ID 56637) were amplified using PCR with SacI and XhoI sites at their extremities to enable ligation into the pmirGLO empty vector (Promega, USA). Binding-region mutations were achieved using a QuikChange Site-Directed Mutagenesis Kit (Stratagene) following the manufacturer’s instructions. Transient transfection of Obs was carried out in 96-well plates with Lipofectamine 2000 (Life Technologies) following the manufacturer’s instruction. The cells were co-transfected with 100 ng luciferase constructs and miRNA mimics or corresponding NCs at a final concentration of 100 nM. The luciferase assays were performed with the dual-luciferase reporter assay system (Promega) following the manufacturer’s instructions. Luminescent signals were quantified using luminometer (Glomax, Promega), and each value from the firefly luciferase construct was normalized with a Renilla luciferase assay.

### Statistical analyses

All numerical data were presented as means±s.d. All statistical analyses were performed with SPSS 13.0. Statistical differences among groups were analysed using one-way analysis of variance. Statistical differences between two groups were determined using the Student’s *t*-test. *P*<0.05 was considered statistically significant.

## Additional information

**How to cite this article:** Zhang, X. *et al*. Cell-free 3D scaffold with two-stage delivery of miRNA-26a to regenerate critical-sized bone defects. *Nat. Commun.* 7:10376 doi: 10.1038/ncomms10376 (2016).

## Supplementary Material

Supplementary Information Supplementary Figures 1-24, Supplementary Table 1, Supplementary Note 1 and Supplementary References.

## Figures and Tables

**Figure 1:**
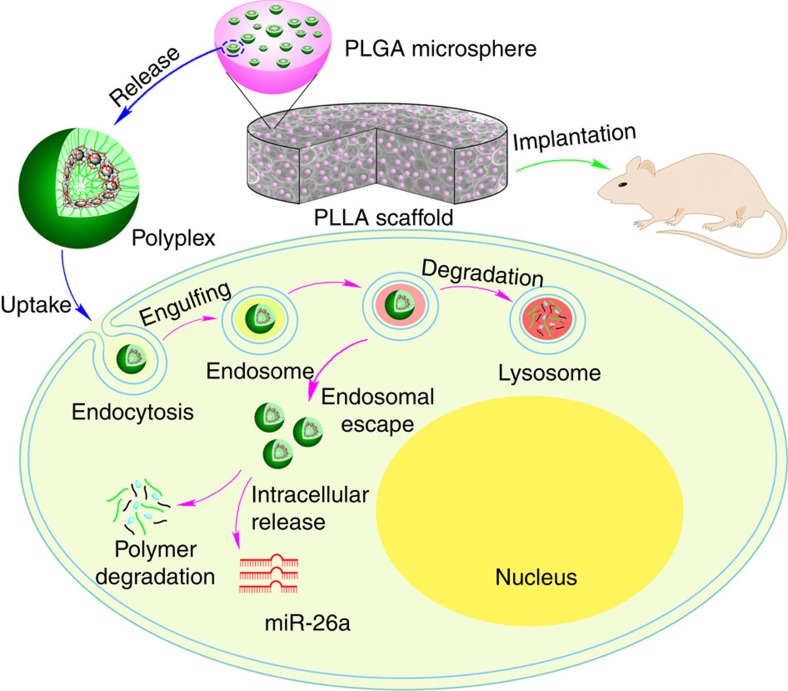
Two-stage delivery of miRNA from PLGA microspheres immobilized on an NF scaffold. HPs and miRNA formed polyplexes in water. The HP/miRNA polyplexes were encapsulated into the PLGA microspheres via the double emulsion method, and the PLGA microspheres containing the HP/miRNA polyplexes were then attached on the PLLA NF scaffold. The PLGA microsphere-incorporated PLLA scaffolds were implanted into mice. The HP/miRNA polyplexes released from the PLGA microspheres (on the PLLA scaffold) could be taken into cells through endocytosis. Intracellular release of miRNA in the cytosol after enzymatic polymer degradation allows its regulation of gene expression.
