# Supplementary material for: Cell-free 3D scaffold with two-stage delivery of miRNA-26a to regenerate critical-sized bone defects
Source: Nat Commun. 2016 Jan 14;7:10376. doi: 10.1038/ncomms10376 (PMC4735608; doi:10.1038/ncomms10376)
Supplement: Supplementary Information — Supplementary Figures 1-24, Supplementary Table 1, Supplementary Note 1 and Supplementary References. [file ncomms10376-s1.pdf]

Supplementary Figures

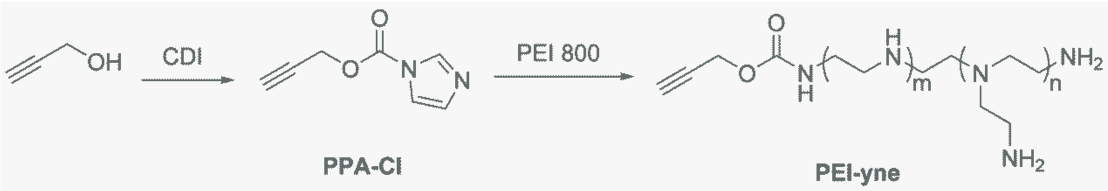

Supplementary Figure 1. Synthesis of PEI-yne. First, propargyl alcohol was reacted with 1,1'-carbonyldiimidazole (CDI) at room temperature to obtain the propargyl ester of carbonylimidazole (PPA-CI). Then, polyethylenimine (PEI, Mw 800 Da) was reacted with PPA-CI to synthesize PEI-yne.

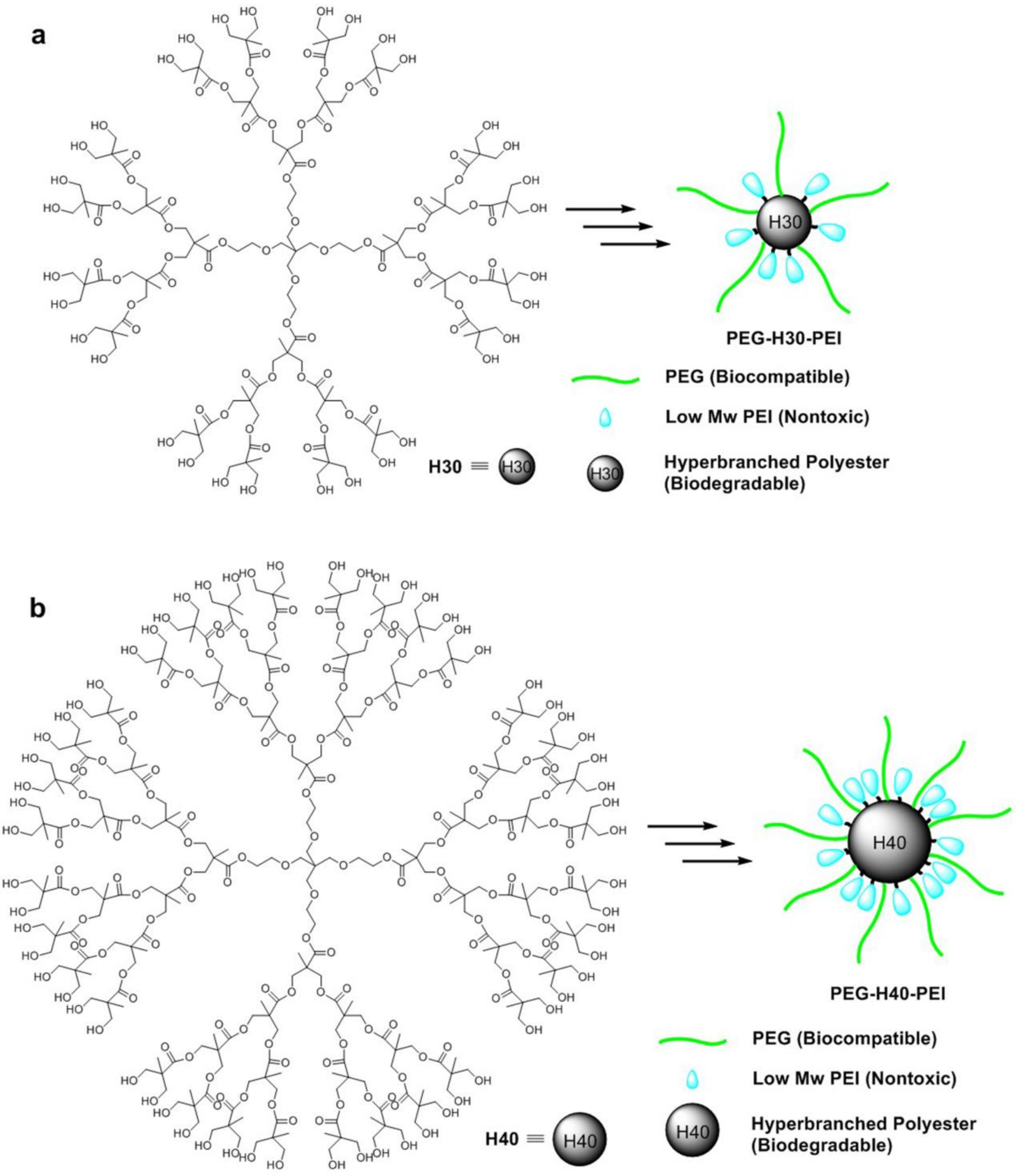

Supplementary Figure 2. Synthesis of hyperbranched polymer PEG-H30-PEI (a) and PEG-H40-PEI (b). The route is the same as that of synthesizing hyperbranched polymer PEG-H20-PEI illustrated in **Scheme S2** (three-step reaction), except H30 (32 hydroxyl) or H40 (64 hydroxyl) is used instead of H20.

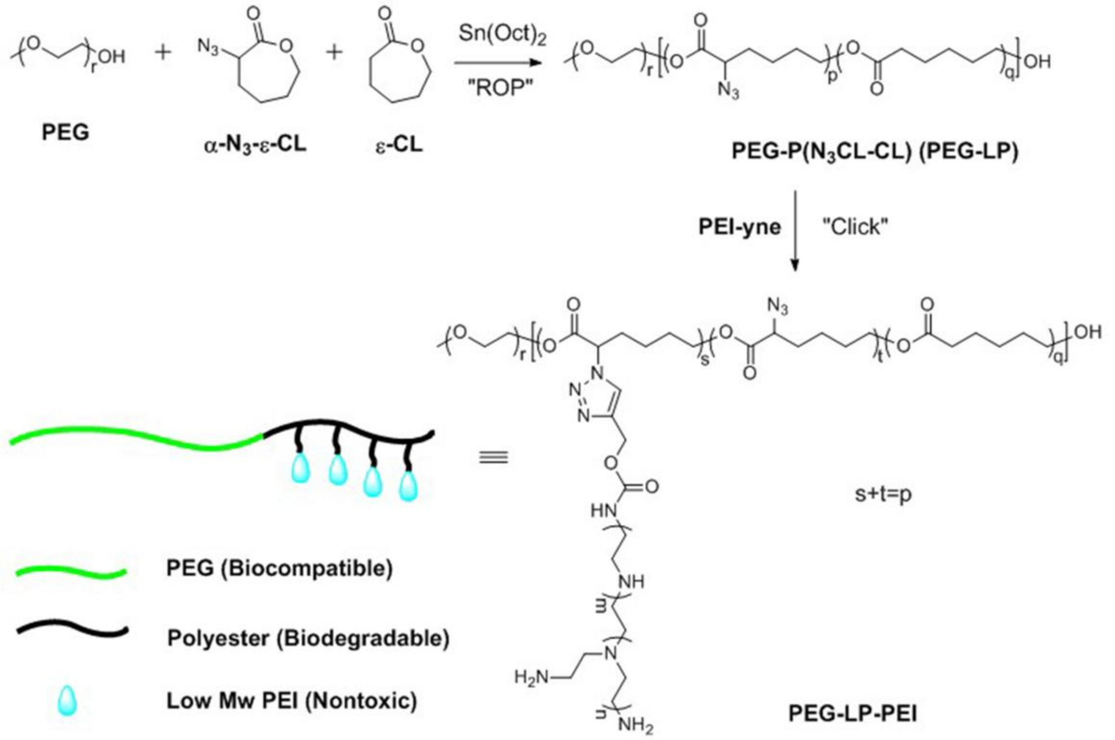

Supplementary Figure 3. Synthesis of linear polymers PEG-LP-PEI. PEG-P(N_3CL-CL) (PEG-LP) was first synthesized by ring-opening polymerization of α-N_3-ε-CL and ε-CL with PEG as an initiator and $Sn(Oct)_2$ as a catalyst. Then, PEG-LP was conjugated with PEI-yne via click chemistry to obtain PEG-LP-PEI.

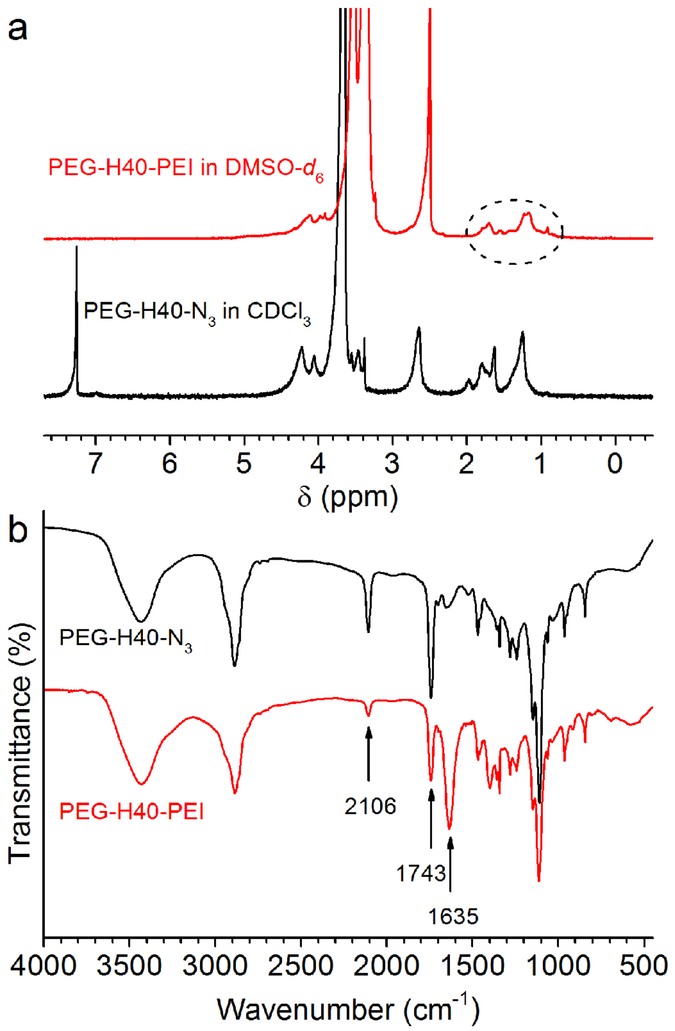

Supplementary Figure 4. ^{1}H NMR spectra (a) and FT-IR spectra (b) of PEG-H40-N_3 and PEG-H40-PEI. The approximate values of molecular weight could be calculated by comparing the characteristic peak integration of Hx, PEI, and PEG in ^{1}H NMR spectrum. Compared with the FT-IR spectrum of PEG-H40-N_3, the height of the characteristic peak at 2106 cm^{-1} for azido groups clearly weakened, the characteristic peak at 1743 cm^{-1} for the carbonyl was still very strong, and a new peak at 1635 cm^{-1} derived from the amino groups of PEI, which indicated that PEI could be conjugated via click chemistry to PEG-H40-N_3 with azido groups and very high grafting ratio, and without degradation of hyperbranched polyester H40. The azido groups on the hyperbranched polyester H40 were difficult to completely react due to the steric hindrance of PEI; thus, there remained residual azido groups after the click chemistry.

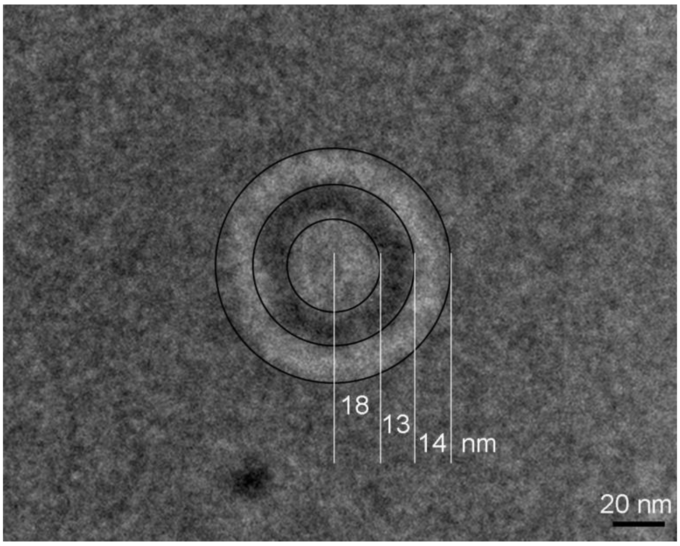

Supplementary Figure 5. The thickness of different layers from the TEM image of HP3 with W-incorporated miRNA. There are two C-O (143 pm) bonds and a C-C (154 pm) bond in each repeating unit of PEG. The bond angles of C-C-O and C-O-C are both ~110º. The length of PEG (Mw 2000) is calculated according to the following equation: $L = (2 \times 143 \times \sin 55 + 154 \times \sin 55) \times 45 = 16$ nm. The thicknesses of both the outer and inner layers are around 16 nm, suggesting that both the outer and inner layers are hydrophilic PEG. The hydrophobic polyester and the miRNA form the spherical shell sandwiched between the outer and inner PEG layers, appearing as a dark ring in the TEM image. Therefore, the combination of HP3 with miRNA results in a 3-layer nanoparticle.

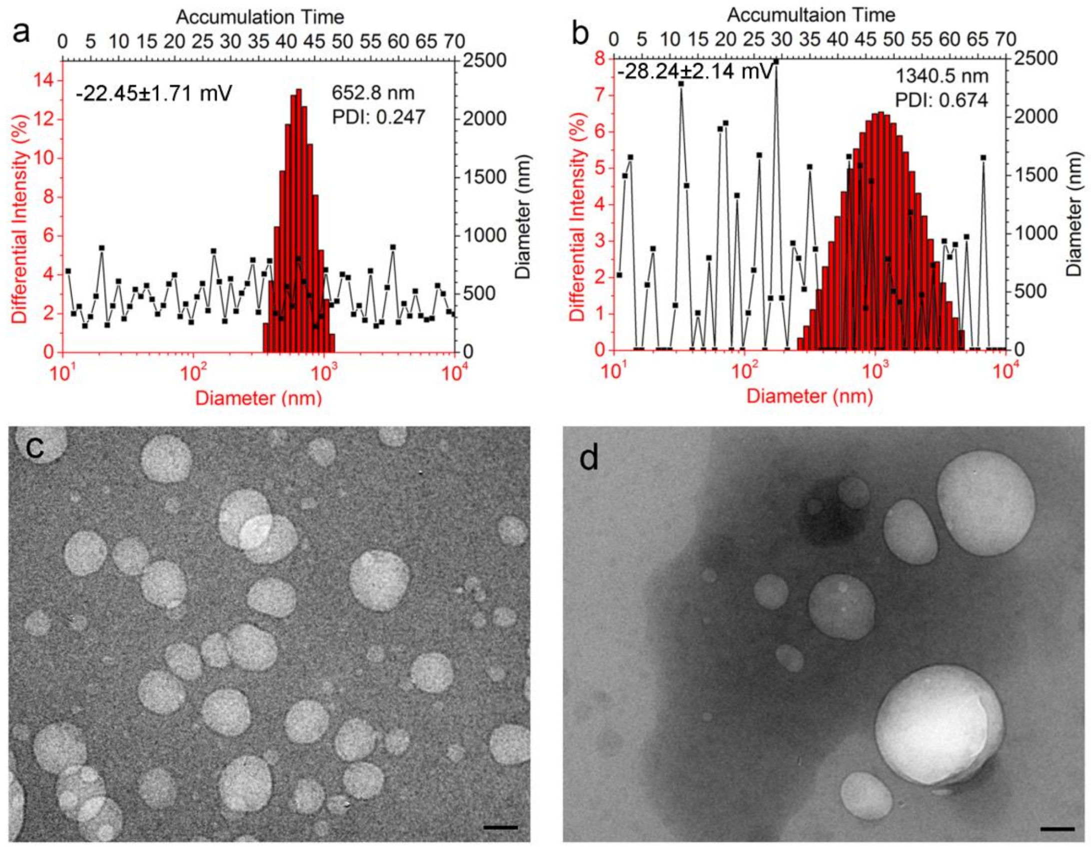

Supplementary Figure 6. Size distribution profile and zeta potential determined by DLS for lipofectamine/miRNA lipoplexes before (a) and after (b) sonication, and micrographs of lipofectamine/miRNA lipoplexes before (c) and after (d) sonication obtained under TEM (scale bar, 200 nm). The lipoplexes became bigger in size, wider in distribution, and more negative in Zeta potential after sonication, indicating that lipoplexes were unstable under sonication.

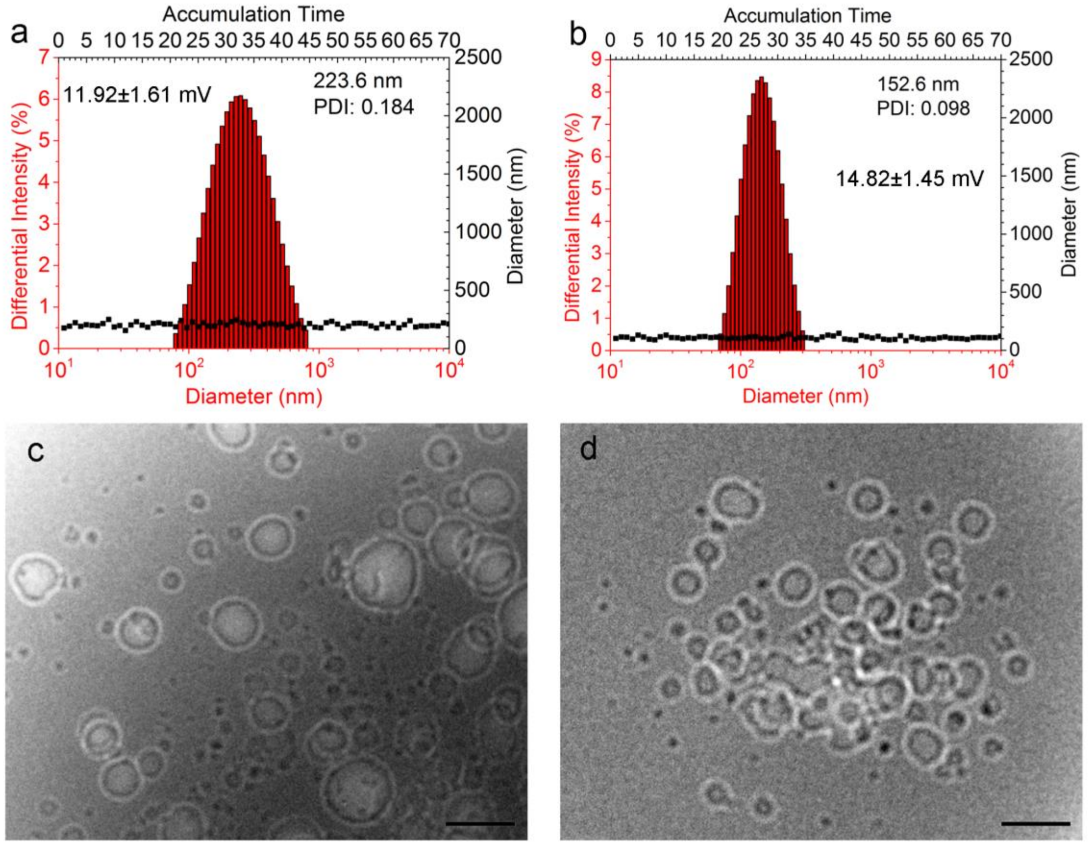

Supplementary Figure 7. Size distribution profile and Zeta potential determined by DLS for the HP3/miRNA polyplexes before (a) and after (b) sonication, and micrographs of the HP3/miRNA polyplexes before (c) and after (d) sonication obtained under TEM (scale bar, 100 nm). The size and the Zeta potential of the HP3/miRNA polyplexes and the PDI did not change significantly under sonication, indicating that the HP3/miRNA polyplexes were very stable. The HP dissolved in water with an average diameter of about 40 nm measured using DLS, which is approximately the same size calculated using the single HP3 molecule (PEI is distributed in a single molecular shell). The HP/miRNA polyplexes have a significantly larger particle size (152 nm measured by DLS) than a HP molecule. The miRNA promoted the formation of double-shelled PEI-miRNA aggregates, where the negatively charged miRNA likely served as electrostatic linker for the positive charged (PEI-containing) HPs.

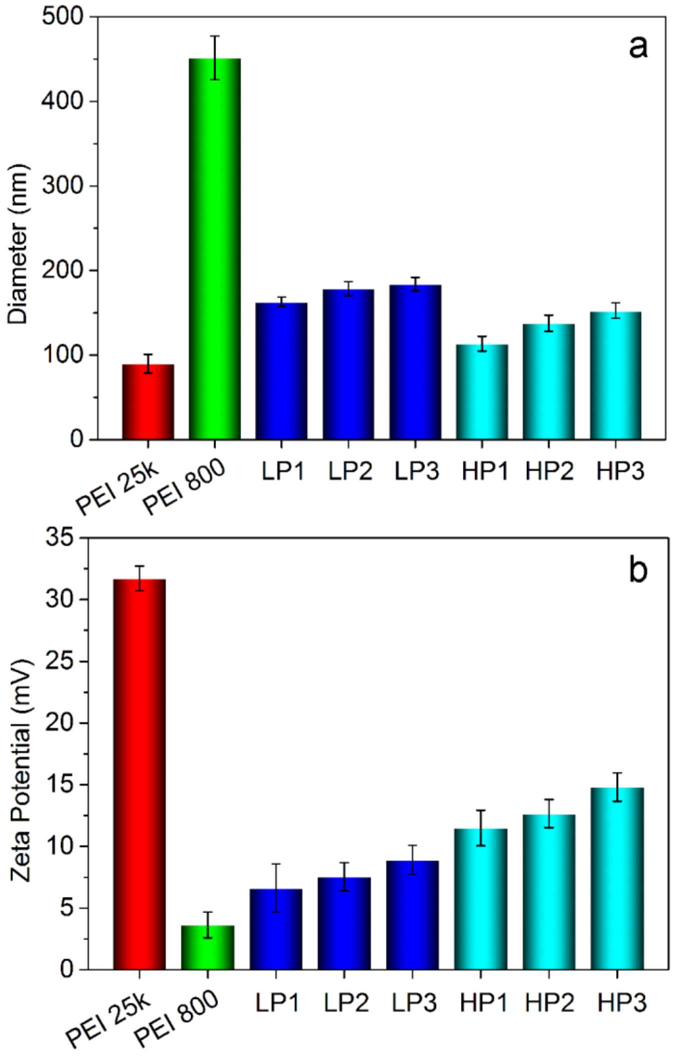

Supplementary Figure 8. Particle size (a) and zeta potential (b) of polymer/RNA polyplexes at a N/P ratio of 10 measured by dynamic light scattering (DLS) at room temperature. 25 kDa PEI could form ~100 nm stable nanoparticles with RNA at high potential, while 800 Da PEI had a larger size with lower potential. The diameters and zeta potential of linear or hyperbranched polymers/RNA polyplexes were between those of 25 kDa PEI and 800 Da PEI.

No.	PLGA MS	miRNA	labeled miRNA	labeled agomir	HP3
1	+	+			+
2	+		+		
3	+			+	
4	+		+		+

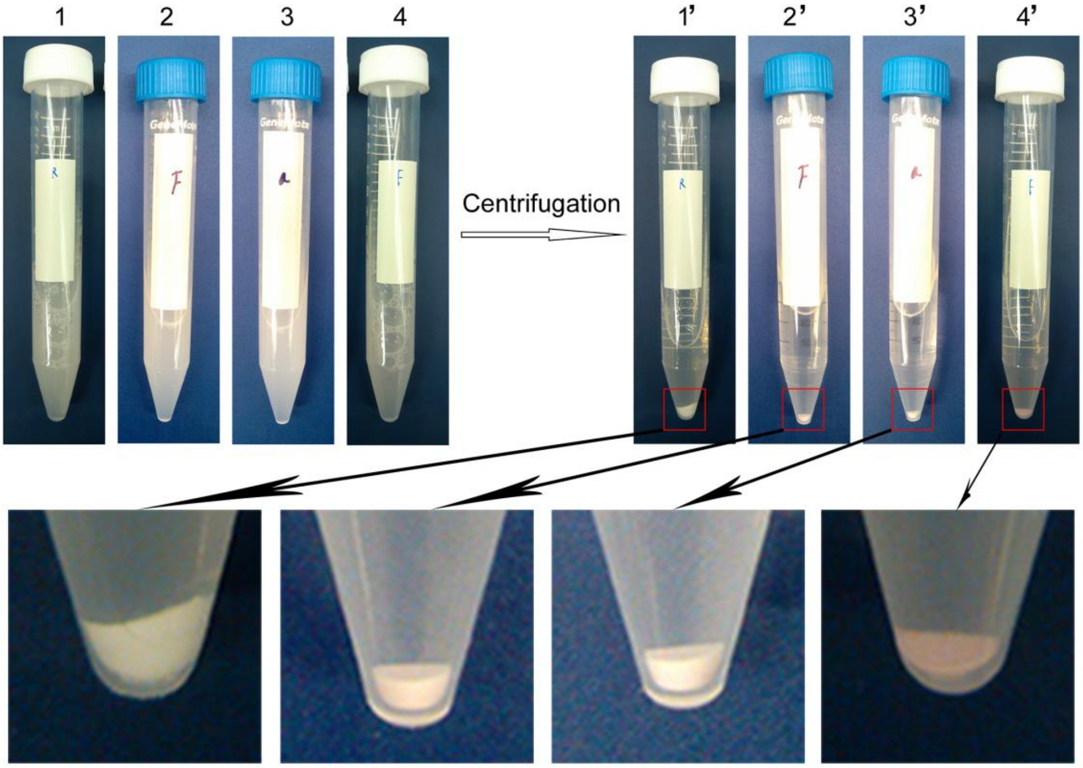

Supplementary Figure 9. The images of PLGA microspheres before (No. 1-4) and after (No. 1'-4') centrifugation. The PLGA microspheres could be centrifuged and the solution was very clear (No. 1'). Naked miRNA (No. 2') and agomir (No. 3') were entrapped into the PLGA microspheres with a low efficiency so that a very weak red color from fluorescence miRNA could be found in the precipitate. However, the HP3/miRNA polyplexes (No. 4') could be entrapped into the PLGA microspheres with a high efficiency, and a very strong red color from fluorescence miRNA could be seen.

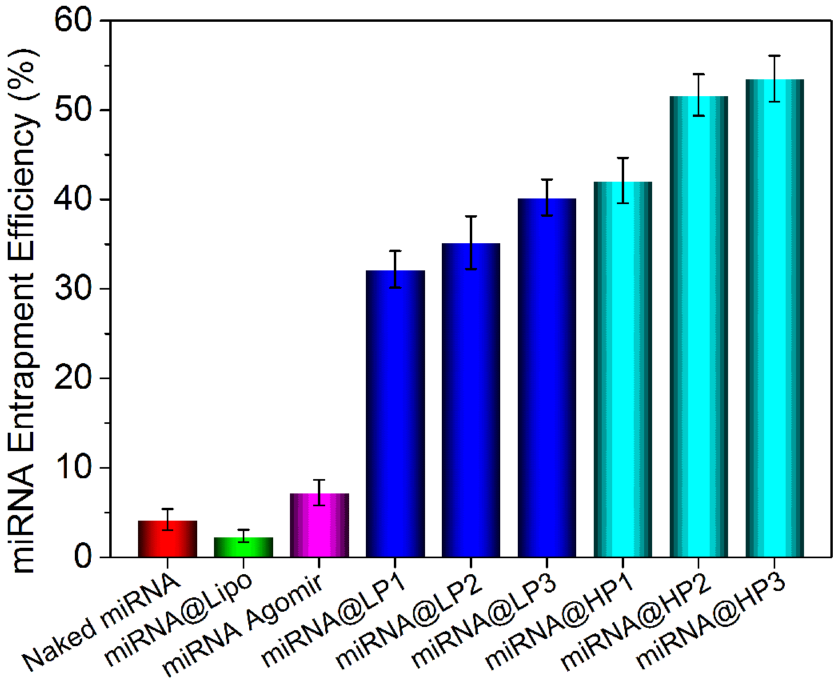

Supplementary Figure 10. miRNA entrapment efficiencies of naked miRNA, lipofectamine/miRNA lipoplexes, miRNA agomir, linear and hyperbranched polymer/miRNA polyplexes into PLGA microspheres. The efficiencies of naked miRNA, lipofectamine/miRNA lipoplexes, and miRNA agomir entrapment were very low (< 10%). However, linear and hyperbranched polymers/miRNA polyplexes had high RNA entrapment efficiencies. The efficiencies of miRNA polyplexes entrapment with hyperbranched polymers (especially HP2 and HP3) were substantially higher than those with linear polymers.

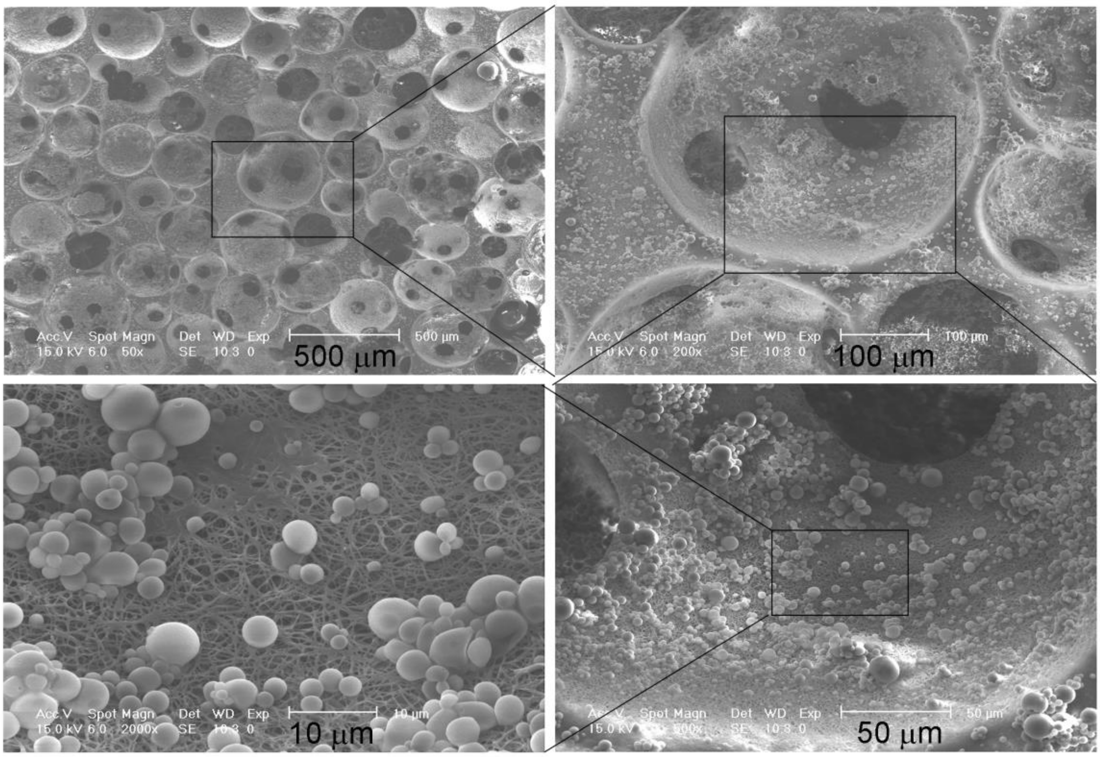

Supplementary Figure 11. SEM images of PLGA microspheres containing the HP3/miRNA polyplexes attaching to a PLLA nano-fibrous scaffold. The disk-shaped nanofibrous PLLA scaffold was 5 mm in diameter and 2 mm in thickness with pore sizes in the range of 250-425 μm. The diameters of PLGA microspheres were ~3 μm. The PLGA microspheres were dispersed on throughout the surface and pores of the PLLA scaffold. The PLLA scaffold retained the nano-fibrous architecture after PLGA microsphere attachment.

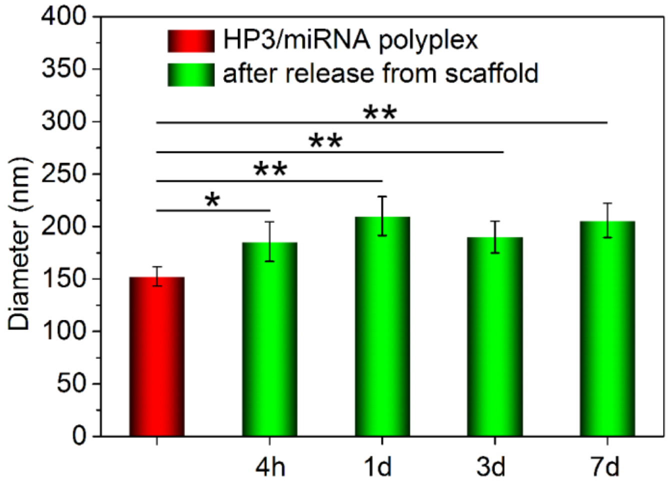

Supplementary Figure 12. Diameters of the HP3/miRNA polyplexes after release from scaffold. After release, the polyplexes had little discernible diameter change. These results indicated that the polyplexes were quite stable even if entrapped within PLGA microspheres and subsequently released from them. * $P<0.05$; ** $P<0.01$.

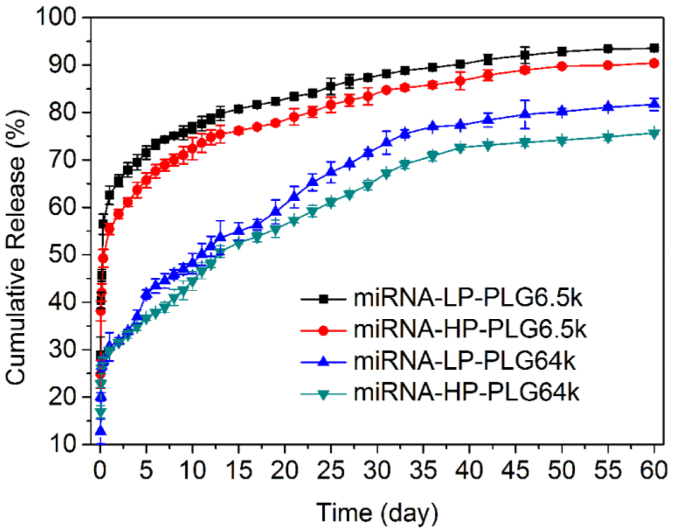

Supplementary Figure 13. Release profile of miRNA from PLGA 6.5k or 64k microspheres containing LP3/miRNA polyplexes or HP3/miRNA polyplexes (PBS, 0.1 M, pH 7.4, 37 ºC). PLGA6.5k MS led to ~60% initial burst release and the total release amount was 90% at the end of two months. PLGA64k MS led to 25% initial burst release and the total release amount was 80% at the end of two months. Therefore, there was approximately 30% additional release from PLGA6.5k MS and 55% additional sustained release from PLGA64k MS after the initial burst release. For PLGA6.5k, the release rate was significantly reduced after 2 weeks or so, but for PLGA64k, the significant slow down uncured after 5 to 6 weeks. The slower degrading PLGA64k therefore resulted in sustained release over a longer time period. The lower molecular weight of PLGA having the shorter polymer chains likely in a looser structure, leading to a higher initial burst release[1]. In addition, the hydrophilicity of PLGA MS increases with decreasing molecular weight of the carboxyl capped PLGA, leading to increased hydrophilic domains[2], which could contribute to the higher initial burst release of the polyplexes. Therefore, the PLGA6.5k MS resulted in higher initial burst release than the PLGA64k MS.

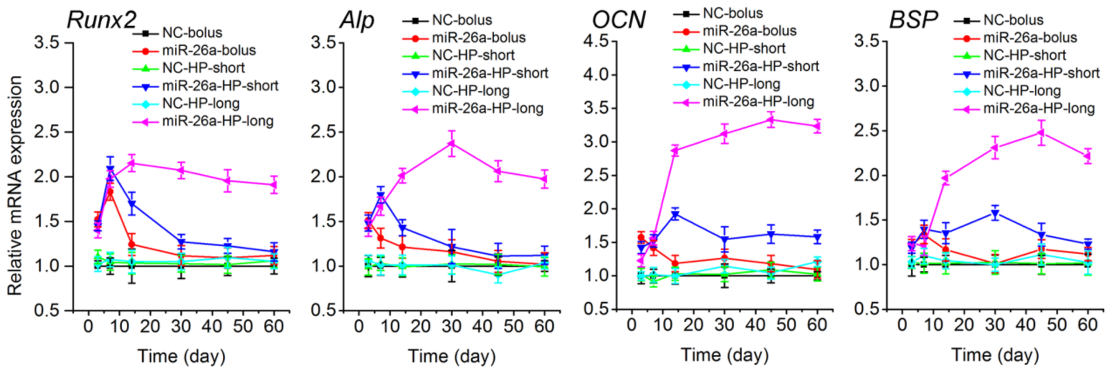

Supplementary Figure 14. Osteogenic genes expression (*OCN*, *Alp*, *Runx2* and *BSP*) over time (at 3, 6, 14, 30, 45, and 60 days) in the implants among the six groups was determined using real-time RT-PCR. All experiments were done in triplicate. n=5 per group. Data are mean±s.d.

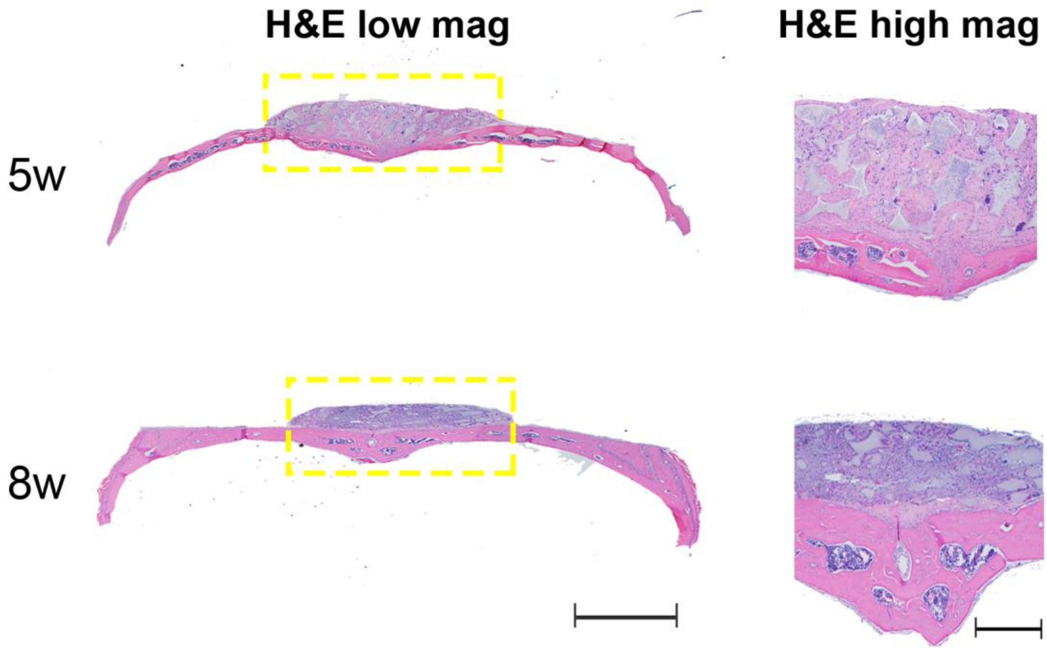

Supplementary Figure 15. The progressive repair of a critical-size calvarial bone defect treated with a long-term miR-26a delivery system was monitored at selected time points including 5 weeks (5w) and 8 weeks (8w) through histological analysis. All experiments were done in triplicate. n=5 per group. Scale bars: 2.0 mm (left) and 200 μm (right).

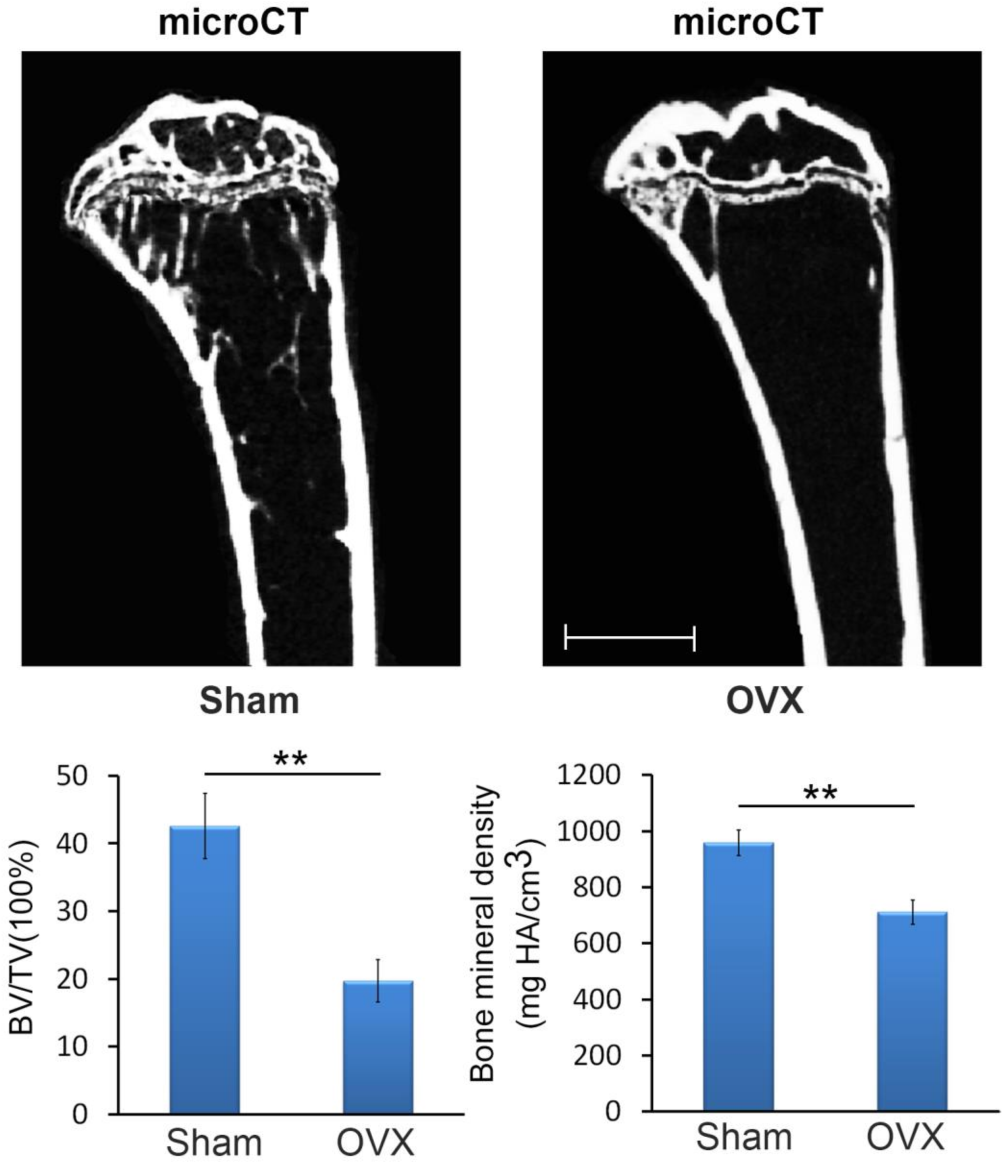

Supplementary Figure 16. MicroCT analysis of proximal tibia in sham- or OVX-operated mice at 8-weeks post-surgery. The microCT revealed that BV/TV was significantly reduced in OVX-operated mice compared to sham-operated mice. ** $P<0.01$. All experiments were done in triplicate. n=5 per group. Data are mean±s.d. Scale bars: 1.0 mm.

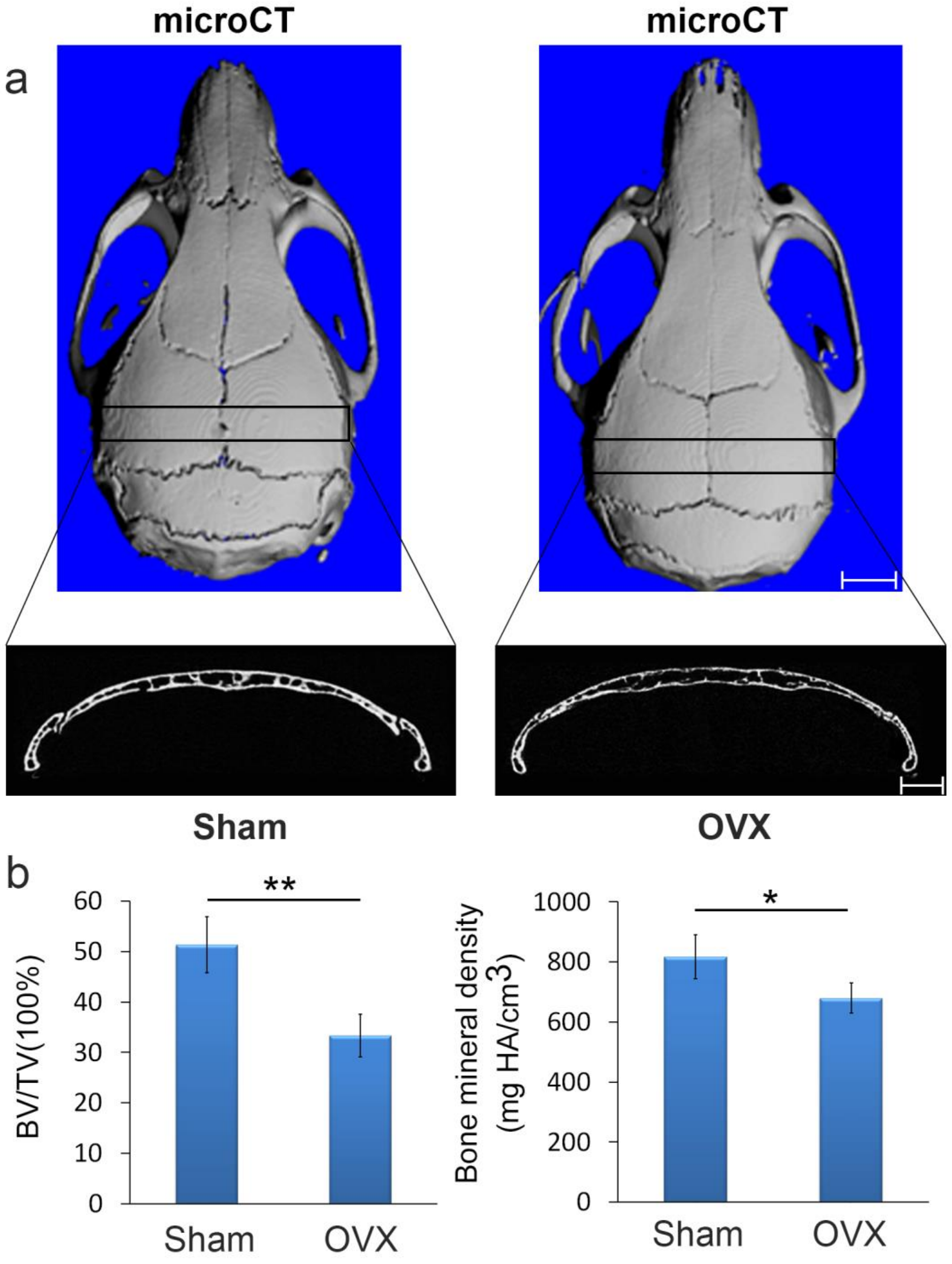

Supplementary Figure 17. (a) MicroCT analysis of calvarial bone of sham- or OVX-operated mice at 8-weeks post-surgery specific to the site as indicated. (b) The microCT revealed that BV/TV and BMD were significantly reduced in OVX-operated mice compared to sham-operated mice. * P<0.05; ** P<0.01. All experiments were done in triplicate. n=5 per group. Data are mean±s.d. Scale bars, 2.0 mm (at the top), 1.0 mm (at the bottom).

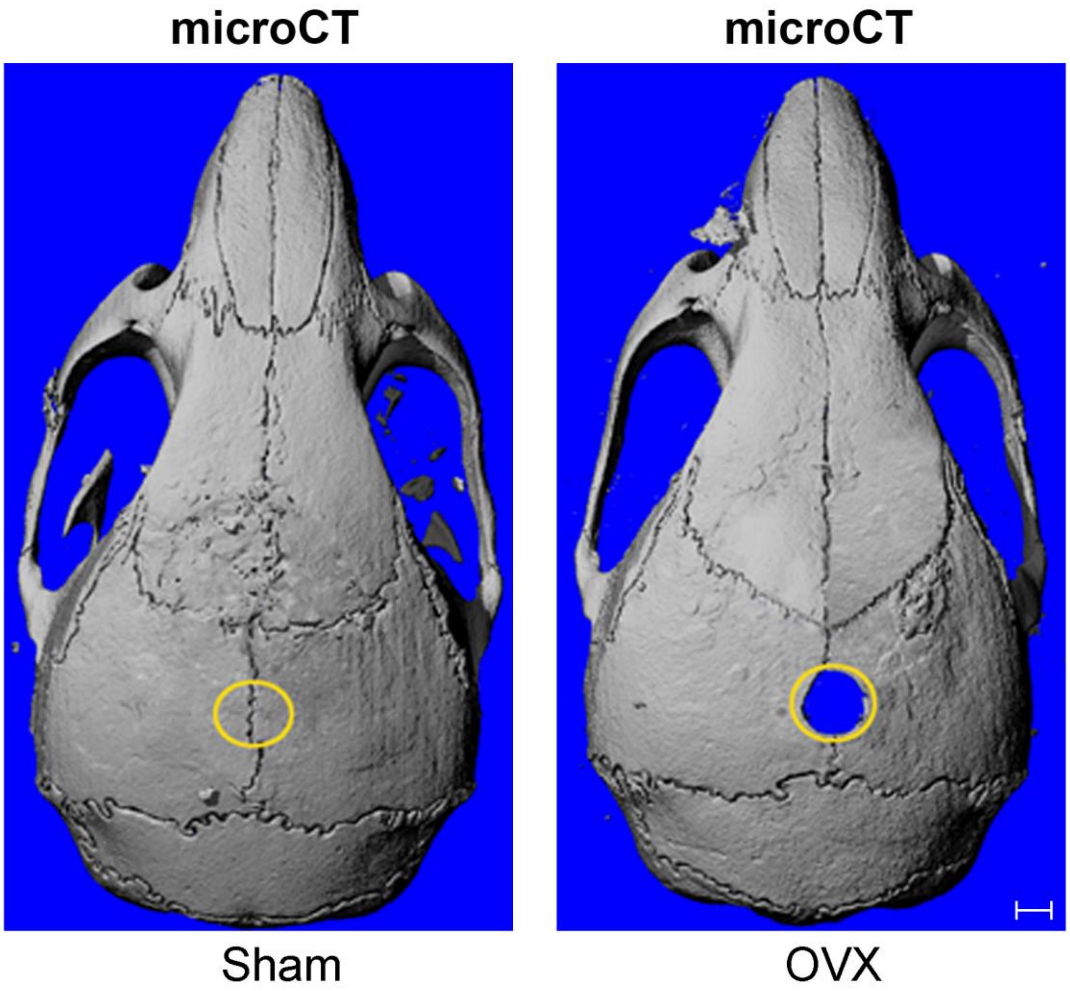

Supplementary Figure 18. A noncritical-size defect (2.5 mm) was generated in the calvarial bone of OVX or sham-operated mice and left empty. MicroCT analysis of calvarial bone at 8 weeks post-surgery. All experiments were done in triplicate. n=5 per group. Scale bars, 1.0 mm.

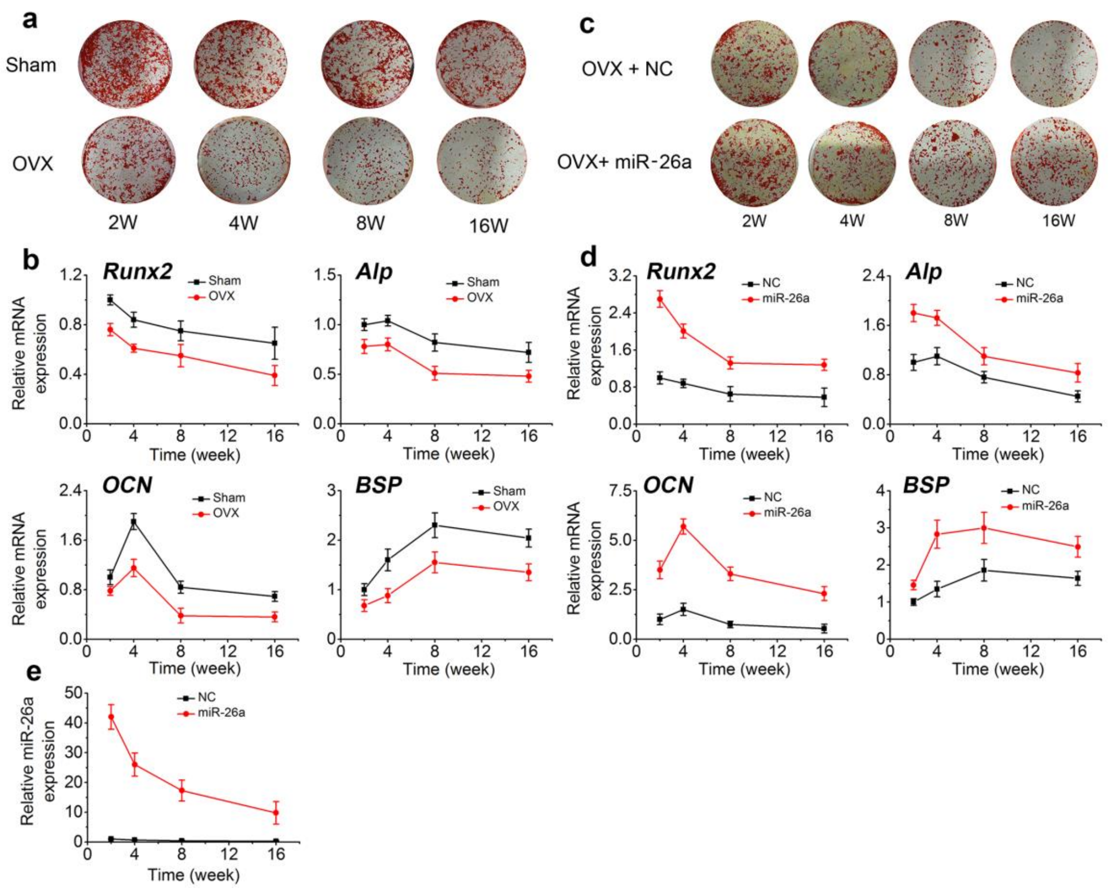

Supplementary Figure 19. miR-26a treatment rescued attenuated bone formation in osteoblasts. (a) Time course changes of bone matrix mineralization deposition within osteoblasts harvested at various time points including 2 weeks (2w), 4 weeks (4w), 8 weeks (8w), and 16 weeks (16w) post-surgery from OVX- or sham-operated mice using Alizarin red staining. (b) Real time RT-PCR analysis of time course changes of osteoblast-associated genes including Runx2, Alp, OCN and BSP mRNA expression levels within osteoblast harvested at 2w, 4w, 8w, and 16w from OVX- or sham-operated mice. (c) Comparison of bone matrix mineralization deposition changes within osteoblast harvested at various times points from OVX-operated mice under treatment with the NC or miR-26a, using Alizarin red staining. (d) Comparison of Runx2, Alp, OCN and BSP mRNA expression levels within osteoblast harvested at various times points from OVX-operated mice under treatment with the NC or miR-26a using real time RT-PCR (e) Real time RT-PCR analysis of miR-26a expression changes within osteoblasts harvested at various time points after treatment with the NC or miR-26a. All experiments were done in triplicate. n=5 per group. Data are mean±s.d.

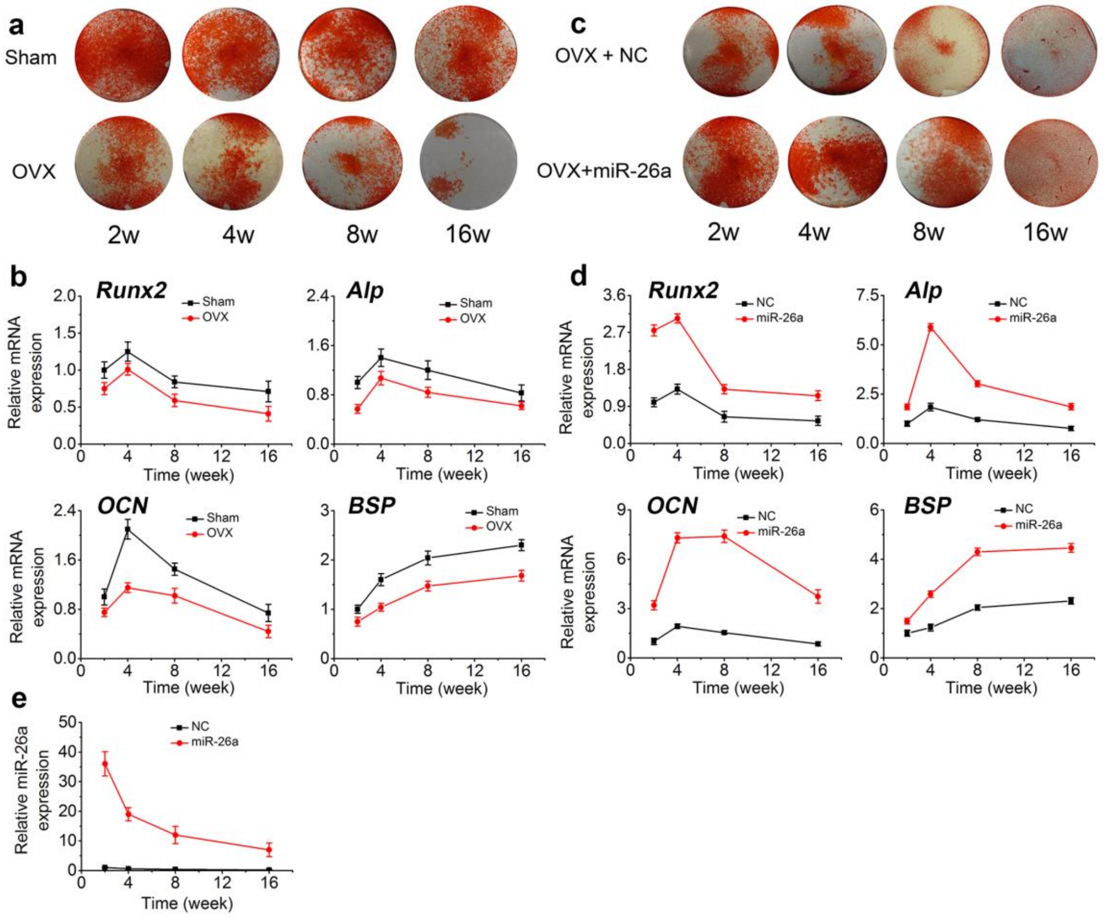

Supplementary Figure 20. miR-26a treatment rescued attenuated bone formation in MSCs. (a) Time course changes in bone matrix mineralization deposition within osteoblasts harvested at various time points including 2 weeks (2w), 4 weeks (4w), 8 weeks (8w), and 16 weeks (16w) post-surgery from OVX- or sham-operated mice using Alizarin red staining. (b) Real time RT-PCR analysis of time course changes of osteoblast-associated genes (Runx2, Alp, OCN and BSP) mRNA expression levels within MSCs harvested at 2w, 4w, 8w, and 16w from OVX- or sham-operated mice. (c) Comparison of changes in bone matrix mineralization deposition within MSCs harvested at various times points from OVX-operated mice under treatment with the NC or miR-26a using Alizarin red staining. (d) Comparison of changes in osteoblast-associated genes (Runx2, Alp, OCN and BSP) mRNA expression levels within MSCs harvested at various times points from OVX-operated mice under treatment with the NC or miR-26a using real time RT-PCR. (e) Real time RT-PCR analysis of miR-26a expression change within MSCs harvested at various times points after treatment with NC or miR-26a. All experiments were done in triplicate. n=5 per group. Data are mean±s.d.

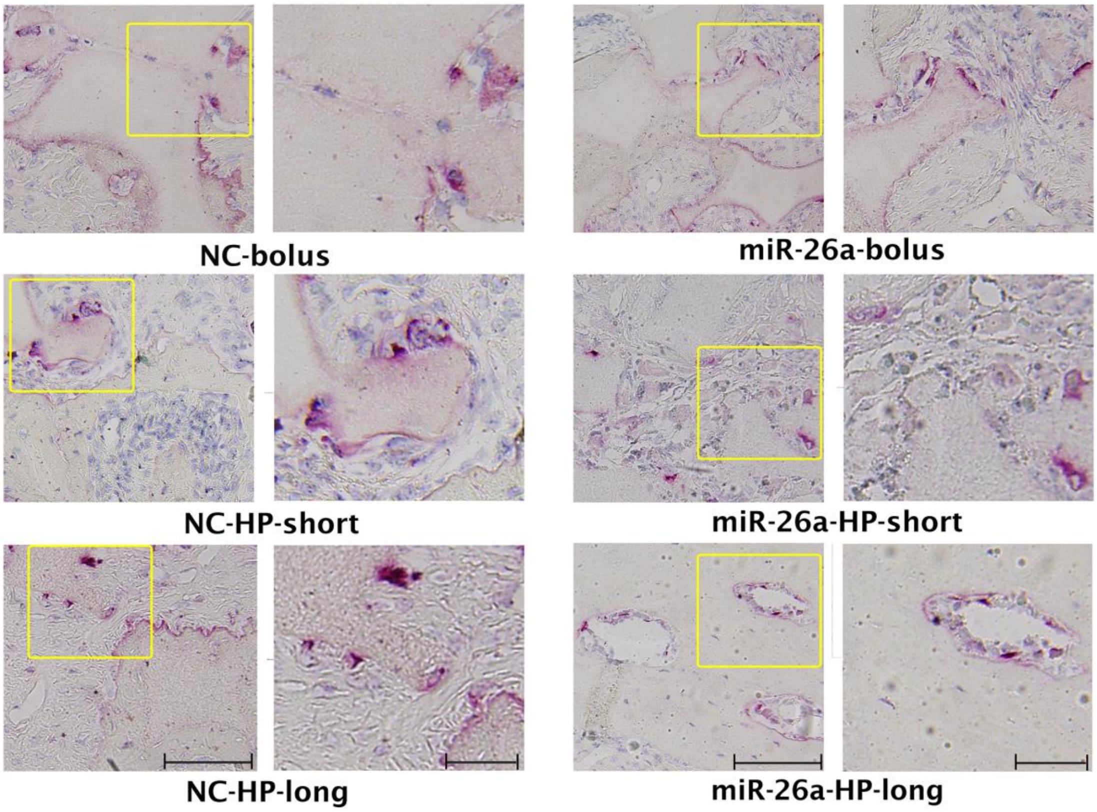

Supplementary Figure 21. Osteoclasts in Osteoporotic (OVX) Mouse Bone Defect Repair in miR-26a Delivering NF PLLA Scaffolds. TRAP staining in histological sections of calvarial bone healing area from OVX-operated mice treated with the six groups (miR-26a-bolus/NC-bolus; miR-26a-HP-short/NC-HP-short; miR-26a-HP-lomg/NC-HP-long). The images at right are higher magnifications. All experiments were done in triplicate. n=5 per group. Scale bars: left, 500 μm; right, 100 μm.

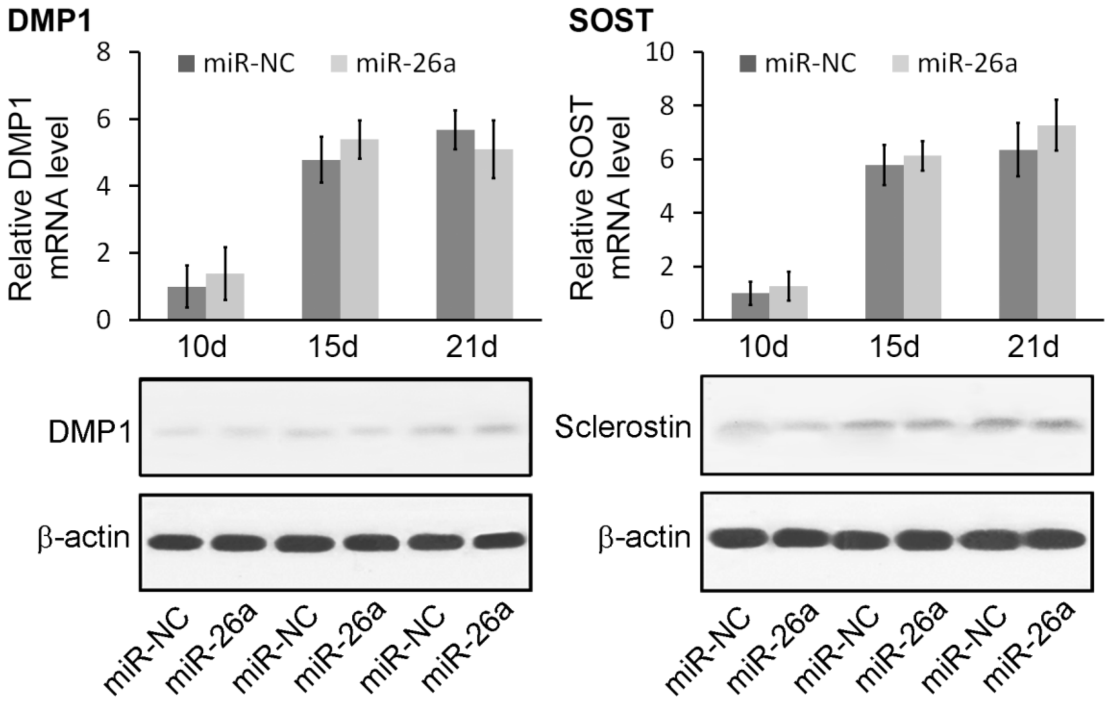

Supplementary Figure 22. The Effect of miR-26a on Osteocyte Markers. The miR-26a treatment did not have statistically significant effect on the expression levels of two typical osteocyte markers during the process of osteoblast differentiation towards osteocyte. Real time RT-PCR and Western blot analysis of time course changes of osteocyte markers (DMP1, SOST) at both mRNA and protein levels upon treatment with miR-26a, anti-miR-26a or their corresponding negative controls after induction for 10, 15 and 21 days. All experiments were done in triplicate. n=5 per group. Data are mean±s.d. The full scans of blots in this figure are provided in Supplementary Fig. 24.

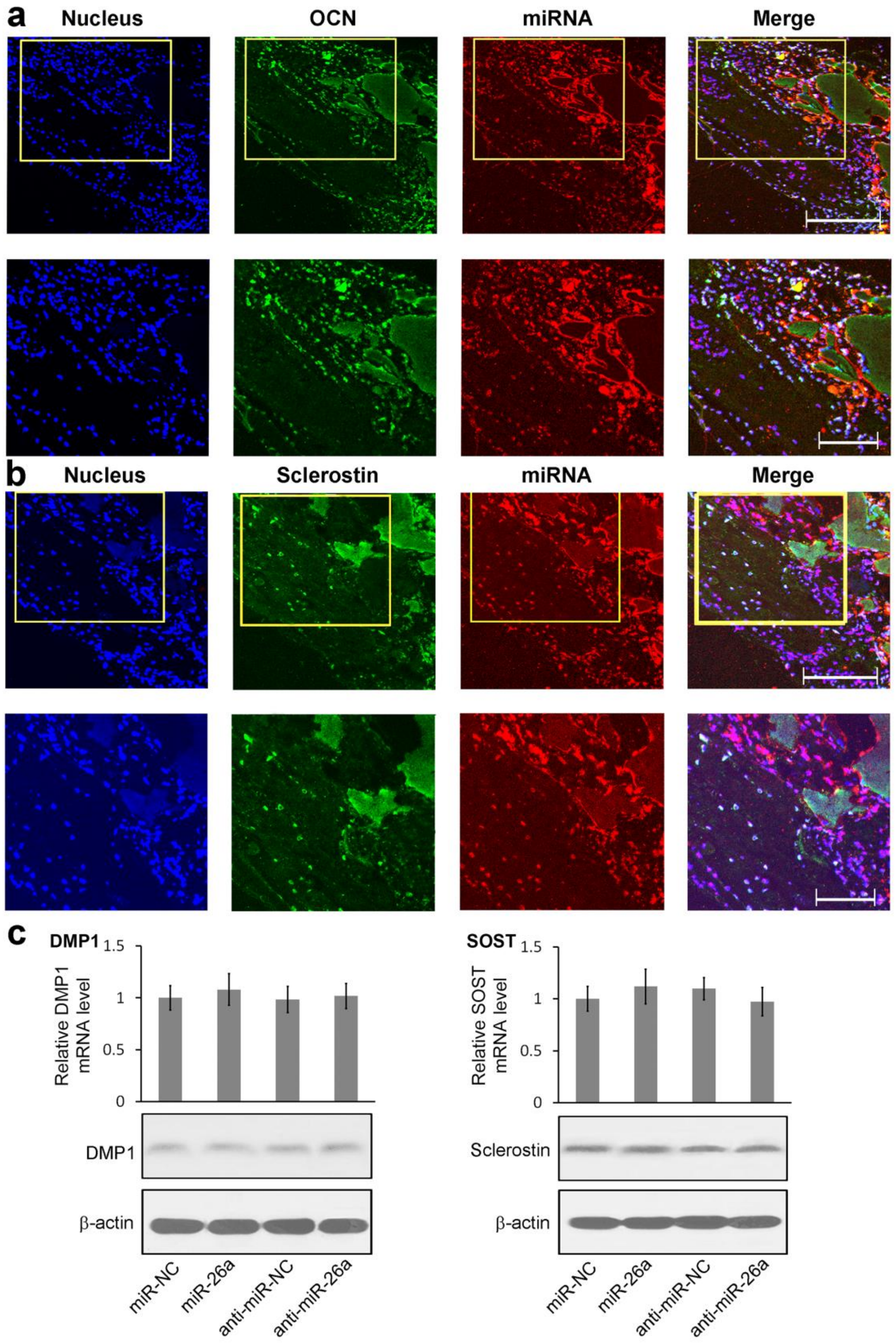

Supplementary Figure 23. miR-26a has no significant effect on osteocyte function. Immunofluorescence staining for OCN (a), Sclerostin (b) and DAPI staining for nuclei in histological sections of the healing calvarial bone. The scale bars are 500 μm (upper panels) and 200 μm (lower panels). (c) Real time RT-PCR and western blot

analyses of osteocyte markers (DMP1, SOST) within osteocytes treated by miR-26a, anti-miR-26a or their negative controls. All experiments were done in triplicate. n=5 per group. Data are mean±s.d. The full scans of blots in this figure are provided in Supplementary Fig. 24.

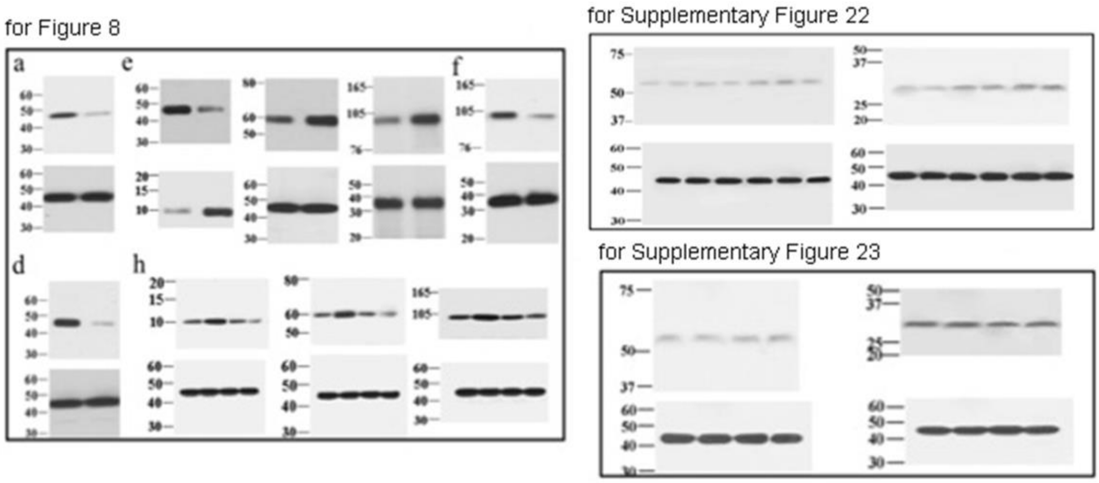

Supplementary Figure 24. Full scans of western blots of Figure 8, Supplementary Figures 22 and 23.

Supplementary Tables

Supplementary Table 1. Synthetic results of linear polymers PEG-PC-PEI and hyperbranched polymers Hx-PEI-PEG

No.	Sample[a]	Element analysis			No. of PEI[b]	W_{PEG} (%)[c]	W_{PEI} (%)[c]	M_n (NMR)	M_n (GPC)	PDI (GPC)	M_n (MALDI-TOF)
		C (%)	H (%)	N (%)							
LP1	PEG-PC$_{16}$-PEI$_{3.3}$	45.06±0.49	7.72±0.24	8.37±0.20	3.3	50.0	26.4	10000	14500	1.33	12800
LP2	PEG-PC$_{34}$-PEI$_{7.7}$	45.33±0.48	7.67±0.28	9.11±0.18	7.7	46.7	28.8	21400	24700	1.24	22500
LP3	PEG-PC$_{71}$-PEI$_{15.9}$	44.92±0.40	7.63±0.25	9.26±0.21	15.9	46.2	29.8	43300	45400	1.36	44300
HP1	H20-PEI$_{2.9}$-PEG$_2$	49.72±0.51	7.90±0.16	7.20±0.25	2.9	39.2	22.7	10200	12800	1.41	11000
HP2	H30-PEI$_{6.5}$-PEG$_5$	50.64±0.52	7.75±0.17	7.16±0.22	6.5	43.7	22.7	22900	27100	1.39	24600
HP3	H40-PEI$_{13.7}$-PEG$_9$	48.64±0.49	7.67±0.18	7.78±0.23	13.7	40.5	24.7	44400	47300	1.29	42500

[a] The number-average molecular weights of PEG in LP1, LP2, and LP3 were 5000, 10000, and 20000 Da, respectively. The number-average molecular weight of PEG in the hyperbranched polymers Hx-PEI-PEG was 2000 Da.

[b] The number of PEI in the polymers was calculated through the content of nitrogen atom determined by element analysis.

[c] The weight ratios of PEG and PEI in the polymers were calculated when the molecular weight was determined from ^{1}H NMR spectra.

Supplementary Notes

Supplementary Note 1. Osteoporotic Mouse Model and Characterization

The osteoporotic mouse model was established two months after ovariectomizing (OVX) female C57BL/6J mice [3] and confirmed by quantification of the bone volume/tissue volume ratio (BV/TV) in the proximal portion of the tibia using microCT. The microCT analysis revealed that BV/TV and BMD were both significantly reduced in OVX operated mice (**Supplementary Fig. 16**).

To determine whether osteoporosis affected bone mass and remodeling in calvaria, we used microCT to examine the 3D architectural parameter changes in the trabecular bone of the osteoporotic calvaria in **Supplementary Fig. 17a** (location indicated). Histomorphometric analysis revealed that the BMD and BV/TV ratio in OVX-operated mice were significantly decreased compared to sham-operated mice (**Supplementary Fig. 17b**). To further detect whether the altered histomorphometric parameters affected bone-healing capacity, a noncritical-size calvarial defect (2.5 mm) was created in the calvarial bone of OVX- or sham-operated mice and left empty to examine the effect of osteoporosis on bone defect healing.

As shown in **Supplementary Fig. 18**, consistent with the decreased histomorphological parameters, bone repair ability was largely attenuated in the OVX-operated calvaria compared to the sham-operated calvaria, which was completely repaired naturally 8 weeks post-surgery. The data indicated that osteoporosis attenuated bone repair capacity in calvaria.

Among various cell populations, mesenchymal stem cells (MSCs) and osteoblasts (Obs) were considered the major contributors to bone regeneration in the defect area.

To determine whether osteoporosis affects osteoblast differentiation or function, we isolated primary calvarial MSCs and Obs from OVX or sham-operated mice at four selected time points after surgery (2w, 4w, 8w and 16w). Under osteogenic conditions, we found that bone matrix mineral deposition was significantly reduced in MSCs and osteoblasts from OVX-operated mice (OVX-MSCs or OVX-Obs) compared to those in sham-operated mice (Sham-MSCs or Sham-Obs). The reduction was shown starting at an early stage (2 weeks post-surgery) and decreased progressively with time during the induced estrogen deficiency period (4w-16w), indicating that the bone formation capacities of MSCs and Obs were impaired under osteoporotic conditions (**Supplementary Figs. 19a&20a**). The impaired osteoblastic activity was confirmed by the changes in the expression levels of osteoblast-associated genes (*Runx2, Alp, OCN* and *BSP*) based on real time RT-PCR analysis (**Supplementary Figs. 19b&20b**). To specifically determine whether miR-26a could function in MSCs and Obs to rescue their attenuated osteoblastic activity under osteoporotic conditions, we treated mouse OVX-MSCs and OVX-Obs with either miR-26a or NC. Real time RT-PCR analysis revealed that intracellular miR-26a expression levels were substantially upregulated by miR-26a treatment (**Supplementary Figs. 19c&20c**). Consistent with the upregulated miR-26a expression, mineralization was appreciably enhanced in miR-26a treated OVX-MSCs or OVX-Obs over NC treated OVX-MSCs or OVX-Obs (**Supplementary Figs. 19d&20d**). This result was corroborated by real time RT-PCR analysis, showing consistent changes in the expression levels of the

osteoblast-associated genes (*Runx2*, *Alp*, *OCN* and *BSP*) (**Supplementary Figs. 19e&20e**).

Supplementary References

1. Yeo, Y. & Park, K.N. Control of encapsulation efficiency and initial burst in polymeric microparticle systems. *Arch. Pharm. Res.* **27**, 1-12 (2004).
2. Heya, T., Okada, H., Ogawa, Y. & Toguchi, H. Factors influencing the profiles of TRH release from copoly (d, l-lactic/glycolic acid) microspheres. *Int. J. Pharm.* **72**, 199-205 (1991).
3. Wang, X.G., Guo, B.S., Li, Q., Peng, J., Yang, Z.J., Wang, A.Y., Li, D., Hou, Z.B., Lv, K., Kan, G.H., Cao, H.Q., Wu, H., Song, J.P., Pan, X.H., Sun, Q., Ling, S.K., Li, Y.H., Zhu, M., Zhang, P.F., Peng, S.L., Xie, X.Q., Tang, T., Hong, A., Bian, Z.X., Bai, Y.Q., Lu, A.P., Li, Y.H., He, F.C., Zhang, G. & Li, Y.X. miR-214 targets ATF4 to inhibit bone formation. *Nat. Med.* **19**, 93-100 (2013).
